# B4GALT1-dependent galectin-8 binding with TGF-β receptor suppresses colorectal cancer progression and metastasis

**DOI:** 10.1038/s41419-024-07028-3

**Published:** 2024-09-04

**Authors:** Tzu-Hui Hsu, Yu-Chan Chang, Yi-Yuan Lee, Chi-Long Chen, Michael Hsiao, Fan-Ru Lin, Li-Han Chen, Chun-Hung Lin, Takashi Angata, Fu-Tong Liu, Kuo-I Lin

**Affiliations:** 1 Genomics Research Center, Academia Sinica, Taipei, Taiwan; 2 Department of Biomedical Imaging and Radiological Sciences, National Yang Ming Chiao Tung University, Taipei, Taiwan; 3 Department of Pathology, Taipei Medical University Hospital, Taipei, Taiwan; 4 Department of Pathology, College of Medicine, Taipei Medical University, Taipei, Taiwan; 5 Institute of Biological Chemistry, Academia Sinica, Taipei, Taiwan; 6 Department of Chemistry, National Taiwan University, Taipei, Taiwan; 7 Institute of Biomedical Sciences, Academia Sinica, Taipei, Taiwan

**Keywords:** Colon cancer, Glycosylation, Metastasis

## Abstract

Transforming growth factor (TGF)-β signaling is critical for epithelial-mesenchymal transition (EMT) and colorectal cancer (CRC) metastasis. Disruption of Smad-depednent TGF-β signaling has been shown in CRC cells. However, TGF-β receptor remains expressed on CRC cells. Here, we investigated whether the cooperation between tumor-associated N-glycosylation and a glycan-binding protein modulated the TGF-β-driven signaling and metastasis of CRC. We showed that galectin-8, a galactose-binding lectin, hampered TGF-β-induced EMT by interacting with the type II TGF-β receptor and competing with TGF-β binding. Depletion of galectin-8 promoted the migration of CRC cells by increasing TGF-β-receptor-mediated RAS and Src signaling, which was attenuated after recombinant galectin-8 treatment. Treatment with recombinant galectin-8 also induces JNK-dependent apoptosis in CRC cells. The anti-migratory effect of galectin-8 depended on β4-galactosyltransferase-I (B4GALT1), an enzyme involved in N-glycan synthesis. Increased B4GALT1 expression was observed in clinical CRC samples. Depletion of B4GALT1 reduced the metastatic potential of CRC cells. Furthermore, inducible expression of galectin-8 attenuated tumor development and metastasis of CRC cells in an intra-splenic injection model. Our results thus demonstrate that galectin-8 alters non-canonical TGF-β response in CRC cells and suppresses CRC progression.

## Introduction

Colorectal cancer (CRC) ranks as the third most diagnosed malignancy and the second leading cause of global cancer-related deaths [1]. More than a third of patients with CRC ultimately develop metastases. A growing number of studies underscore the pivotal role of genetic factors in the development and progression of CRC. Studies on familial adenomatous polyposis and Lynch syndrome revealed that distinct CRC subtypes are caused by different types of genomic alterations. These include germline mutations of DNA mismatch repair genes, which lead to microsatellite instability (MSI) in Lynch syndrome, or inherited defects and mutations of *APC*, *KRAS*, and *TP53* in familial adenomatous polyposis, which are microsatellite stable (MSS) [2, 3].

Transforming growth factor (TGF)-β signaling, a pivotal pathway in human cancer, governs diverse biological processes [4]. TGF-β binds to type II TGF-β receptor (TβRII), activating type I TGF-β receptor (TβRI) through phosphorylation and initiating signaling. In normal epithelium, TGF-β functions as a tumor suppressor, which triggers Smad2/Smad3 activation and culminates in cell growth arrest and apoptosis [5]. In CRC, TGF-β plays an important role in the process of epithelial-mesenchymal transition (EMT) in the advanced stages. TGF-β-driven metastasis involves Smad-independent pathways, such as Src/RAS, JNK, PI3K/Akt, p38, and NF-κB. [6, 7]. The effect of TGF-β signaling alternation extends to regulating cell adhesion, motility, and extracellular matrix composition [8]. The mechanism by which TGF-β signaling switches from anti- to pro-oncogenic activity remains poorly understood, but genetic alterations of TGF-β signaling components (e.g., TβRI and TβRII) or the absence of Smad4-mediated signal transduction are features of genetically heterogeneous CRC [9–11].

Galectins are a group of lectins that contain one or two carbohydrate recognition domains (CRDs). Galectins can interact with β-galactoside and induce a variety of cellular effects [12]. In humans, 12 galectins have been shown to influence tumor progression by rewiring intracellular and extracellular signaling circuits [13]. For example, galectins 3 and 9 form lattice networks with glycoproteins in cancer cells [14–16]. Abnormal glycosylation in several types of cancers, including CRC [17], modulates cell adhesion, signaling, stemness, and invasiveness [18, 19]. In addition, aberrant N-glycosylation reduces TGF-β binding, leading to a deficiency in TGF-β signaling [20]. However, the roles of glysosylation and galectin functions in CRC remain unclear.

We here established a connection between the anti-malignant effect of galectin-8 and increased B4GALT1 expression in CRC cells. The underlying mechanism involves an interaction between galectin-8 and B4GALT1-galactosylated TβRII, and subsequent inhibition of TGF-β/TβRII binding to downregulate non-canonical signaling activity. Furthermore, recombinant galectin-8 induces apoptosis of CRC cells via JNK activation. Thus, increasing galectin-8 levels could potentially be used as a therapeutic strategy for CRC.

## Materials and methods

### Cell lines

HCoEpi cells were purchased from ScienCell Research Laboratories, Inc. (Carlsbad, CA, USA). CRC cell lines, including DLD1, HT29, H3347, CX1, SW1116, SW48, SW480, LS123, SKCO1, HCT116, SW620, and FHC cells, were purchased from the American Type Culture Collection (ATCC). HCoEpi cells were grown in colonic epithelial cell medium (Cat. No. 4101, ScienCell) supplemented with 2% fetal bovine serum (FBS, Cat. No. 0010, ScienCell), 5% (v/v) of epithelial cell growth supplement (EpiCGS, Cat. No. 4152, ScienCell) and penicillin (100 U/mL)/streptomycin (100 μg/mL) (Cat. No. 0503, ScienCell). All other cell lines were grown in RPMI1640 (Cat. No. 11875, Gibco, Thermo Fisher Scientific, MA, USA), supplemented with 10% FBS (Cat. No. 26140079, Gibco) and penicillin (100 U/mL)/streptomycin (100 μg/mL) (Cat. No. 15140148, Gibco). Cells were incubated at 37 °C under an atmosphere containing 5% CO_2_ and tested for mycoplasma contamination using EZ-PCR^TM^ Mycoplasma Detection Kit (Cat. No. SKU: 20-700-20, Sartorius).

### Mice

Eight-week-old NOD-SCID mice (male) and 8- to 9-week-old C57B/6 mice (male) were purchased from LASCO (Taiwan) and the National Laboratory Animal Center (Taipei, Taiwan), respectively. All mice used were housed in the animal facilities at Experimental Animal Facilities of Academia Sinica (Taipei, Taiwan). All animal experimental procedures were approved by the Institutional Animal Care & Utilization Committees of Academia Sinica.

### Patients

Fifty-seven samples derived from CRC patients were obtained from Wan-Fang Hospital and Taipei Medical University Hospital from 1998 to 2005. Informed consent was obtained from each patient. Approval for use of the tumor collections was provided by the Institutional Review Boards of Taipei Medical University (WFH-IRB-99049).

All other materials and methods, including uncropped immunoblotting images, were described in Supplemenary Materials and Methods.

## Results

### Knockdown of *LGALS8* increases migratory activity of CRC cells

**Fig. 1:**
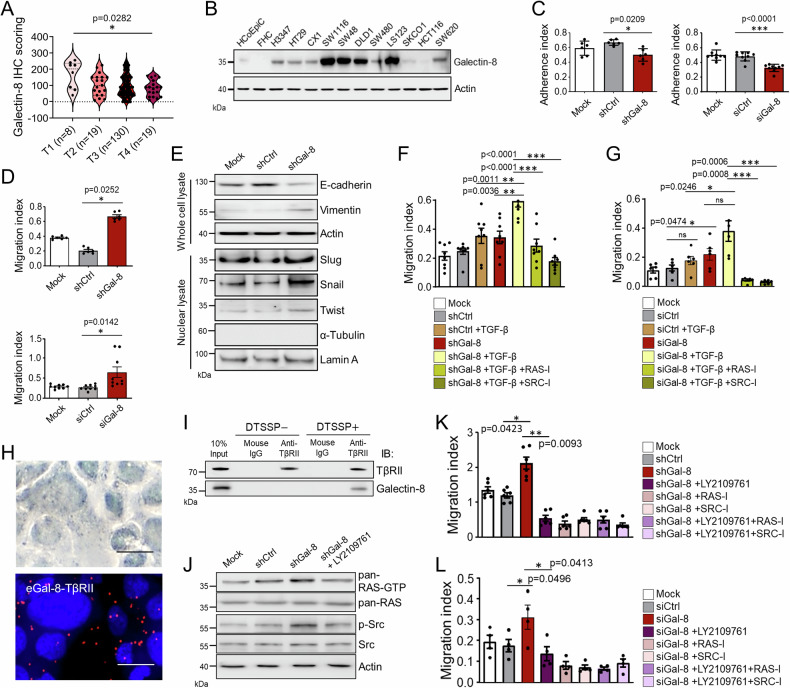
Knockdown of *LGALS8* potentiates the migratory characteristics of TGF-β responsive HT29 cells by increasing RAS and Src activities. **A** Scoring results from IHC staining of galectin-8 in tumor tissues from CRC patients classified by T-classification. **B** Western blotting shows the expression of galectin-8 protein in various CRC cells, FHC and normal human intestinal epithelial cells (HcoEpi). Cell adhesion activities measured 6 h after seeding (**C**) and migration activities measured 24 h after seeding (**D**) of HT29 cells expressing shCtrl or shGal-8 (top) or transfected with a mixture of three different siRNAs targeting galectin-8 (siGal-8) or a scrambled control siRNA (siCtrl) (bottom) were analyzed by xCELLigence RTCA. **E** Western blotting shows the expression of EMT markers in the whole cell lysate and nuclear lysate isolated from the mock-treated, shCtrl-expressing, and shGal-8-expressing HT29 cells. Actin and Lamin A serves as the whole cell lysate and nuclear lysate loading control, respectively. α-tubulin serves as the negative control of nuclear lysate preparation. Cell migration activities of shCtrl- and shGal-8-expressing HT29 cells (**F**) or siCtrl- and siGal-8-transfected HT29 cells (**G**) in the presence or absence of TGF-β (10 ng/mL), the pan-RAS inhibitor lonafarnib (RAS-I, 100 μM), and the Src inhibitor dasatinib (SRC-I, 30 μM), assessed by xCELLigence RTCA at 48 h after seeding. **H** Proximity ligation assay (PLA) to determine the interaction of endogenous galectin-8 with TβRII in HT29 cells. Scale bar = 10 μm. **I** HT29 cells were treated with or without DTSSP crosslinker before cell lysis. Co-immunoprecipitation (Co-IP) was used to examine the interaction of galectin-8 and TβRII by IP of lysates with anti-TβRII antibody or isotype control mouse IgG, followed by immunoblotting (IB) with the indicated antibodies. **J** Western blotting shows the expression of activated, phosphorylated, and total proteins of RAS or Src in mock-treated, shCtrl, shGal-8 alone or with LY2109761 treated HT29 cells. Actin serves as the loading control. Migration activities of HT29 cells expressing the indicated shRNA (**K**) or siCtrl- and siGal-8-transfected HT29 cells (**L**) and/or treated with TGF-β signaling antagonist LY2109761 (10 µM), RAS-I (100 μM), and SRC-I (30 μM) for 48 h. Results in **C**, **D**, **F**, **G**, **K**, **L** are shown as mean ± SD (3 biological replicates with technical duplicates in **C** (left), **D** (top), **G** and **K**; 3 biological replicates with technical tripilicates in **C** (right) and **D** (bottom); 3 biological replicates with technical quintuplicates in **F**; 2 biological replicates with technical duplicates in **L**). Statistical tests were calculated by one-way ANOVA for A and *t*-test for **C**, **D**, **F**, **G**, **K**, **L**. ns not significant.

We first examined galectin expression in CRC cells, immortalized normal fetal human colon cells (FHC), and normal human colorectal epithelial (HCoEpi) cells. Our results showed the elevated galectin-8 mRNA level in several CRC cell lines (Supplementary Fig. S1A). Results from immunohistochemistry (IHC) staining with clinical CRC samples showed that galectin-8 expression is downregulated during CRC tumor progression, with a significant difference between stages T1 and T4 (Supplementary Fig. S1B and Fig. 1A). Similarly, more aggressive CRC lines [21], such as SKCO1 and HCT116, showed lower galectin-8 expression than the other less aggressive CRC cells (Fig. 1B). The aggressiveness of DLD1, HT29, and HCT116 cells was also verified by the migratory activity assays (Supplementary Fig. S1C). Next, we examined the function of galectin-8 in CRC cells by targeting galectin-8 with two different short hairpin RNAs (shRNAs) or a mixture of multiple small inferfering RNAs (siRNAs). The knockdown efficiencies were confirmed in DLD1 and HT29 cells (Supplementary Fig. S1D–G). It is noted that knockdown of *LGALS8* by shGal-8 #2 (hereafter referred to as shGal-8) in HT29 cells reduced adhesion (Fig. 1C, left) and increased migration (Fig. 1D, upper). Similarly, transfection of multiple siRNAs decreased adhesion (Fig. 1C, right) and increased migratory activity (Fig. 1D, lower) in HT29 cells. Knockdown of *LGALS8* with shGal-8 (Fig. 1E) or multiple siRNAs (Supplementary Fig. S1H) also correlated with reduced E-cadherin and increased EMT-promoting proteins, including Vimentin, and nuclear Slug, Snail, and Twist. TGF-β signaling promotes tumor progression [6, 8]. We therefore investigated *LGALS8* knockdown effect on HT29 cells in response to TGF-β. We found that TGF-β treatment exacerbated the migration of HT29 cells following the knockdown of *LGALS8* by shGAl-8 (Fig. 1F); similar results were observed when the cells were transfected with multiple siRNAs (Fig. 1G). Our results thus suggest that the absence of galectin-8 in CRC cells promoted EMT and elevated TGF-β-mediated metastasis.

Mutations of TβRII were found in a majority of CRC samples, which may cause the dysfunctional TGF-β signaling [10, 11]. Because galectin-8 binds to glycoproteins, we hypothesized that it might recognize the glycan moiety on TβRII and thereby regulate TβRII-mediated non-Smad signaling in CRC cells. Indeed, galectin-8 was found to co-localize with TβRII on HT29 cells by proximity ligation assay (PLA) (Fig. 1H). A co-immunoprecipitation (co-IP) assay using HT29 cell lysates confirmed the presence of galectin-8 in anti-TβRII immunoprecipitates (Fig. 1I). Furthermore, *LGALS8* depletion enhances RAS and Src activation (Fig. 1J), the downstream non-SMAD mediators of TGF-β-induced pathways [22]. Consistently, TGF-β receptor antagonist LY2109761 [23] suppresses RAS and Src activation (Fig. 1J). HT29 produced TGF-β (Supplementary Fig. S1I). We validated that the addition of LY2109761, RAS inhibitor lonafarnib (RAS-I) or Src inhibitor dasatinib (SRC-I) abolished the increased migratory activity by *LGALS8* silencing with shRNA and multiple siRNAs, regardless of the treatment with TGF-β (Fig. 1F, G, K, L).

**Fig. 2:**
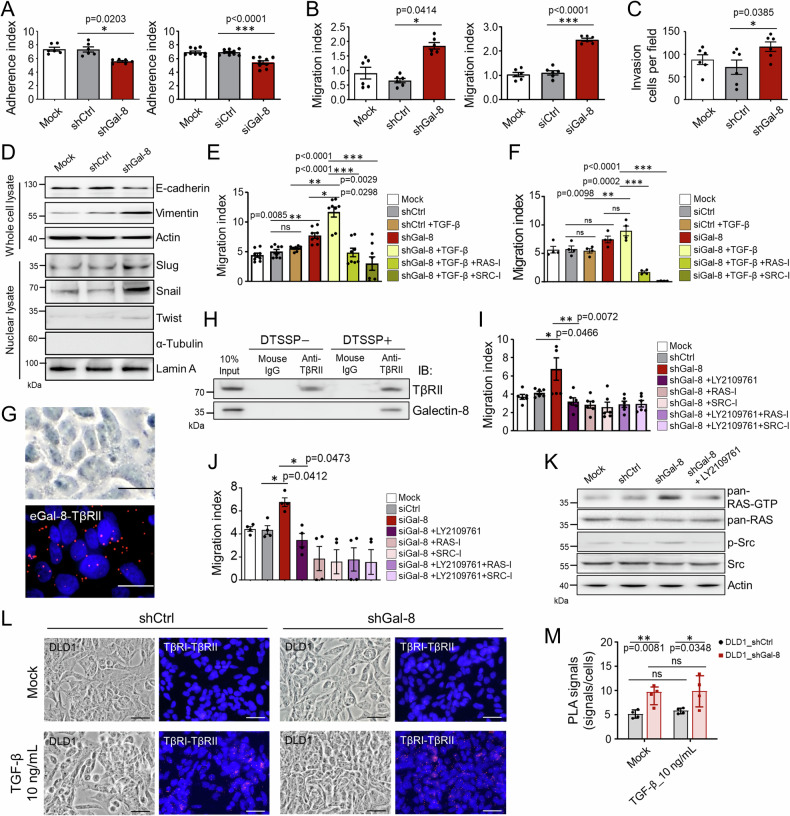
Knockdown of *LGALS8* promotes migratory characteristics and ameliorates the resposnes to TGF-β in DLD1 cells. **A**, **B** Cell adhesion activities measured at 6 h after seeding (**A**) and migration activities measured at 48 h after seeding of DLD1 cells expressing shCtrl or shGal-8 (left) or transfected with siGal-8 or siCtrl (right) were analyzed by xCELLigence RTCA. **C** Cell invasion activities of DLD1 cells expressing shCtrl or shGal-8 were analyzed 24 h after seeding by xCELLigence RTCA. **D** Western blotting shows the expression of EMT markers in mock-treated, shCtrl-expressing, and shGal-8-expressing DLD1 cells. Actin and Lamin A serves as the whole cell lysate and nuclear lysate loading control, respectively. α-tubulin serves as the negative control of nuclear lysate preparation. Cell migration activities of shCtrl- and shGal-8-expressing DLD1 cells (**E**) or siCtrl- and siGal-8-transfected DLD1 cells (**F**) in the presence or absence of TGF-β (10 ng/mL), RAS-I, (100 μM), and SRC-I (30 μM) evaluated by xCELLigence RTCA at 48 h. **G** PLA determining the interaction of endogenous galectin-8 with TβRII in DLD1 cells. Scale bar = 10 μm. **H** DLD1 cells were treated with or without DTSSP crosslinker before cell lysis. Co-immunoprecipitation (Co-IP) showing the interaction of galectin-8 and TβRII by IP of lysates with anti-TβRII antibody and isotype control mouse IgG, followed by immunoblotting (IB) with the indicated antibodies. Migration activities of DLD1 cells expressing indicated shRNA (**I**) or transfected with siRNAs (**J**) and/or with LY2109761 (10 µM), RAS-I, (100 μM), and SRC-I (30 μM) for 48 h. **K** Western blotting shows the expression of activated, phosphorylated, and total proteins of RAS or Src in mock-treated, shCtrl, shGal-8 alone or with LY2109761 treatment in DLD1 cells. Actin serves as the loading control. PLA images (**L**) and statistical analysis of PLA signal quantification (**M**) show the effect of endogenous galectin-8 on the interaction of TβRI with TβRII. DAPI staining was used to label cell nuclei. Scale bar = 20 µm. Results in **A**, **B**, **C**, **E**, **F**, **I**, **J**, **M** are shown as mean ± SD (3, 3, 3, 4, 2, 3, and 2 biological replicates with technical duplicates in **A** (left), **B**, **C**, **E**, **F**, **I**, **J**, respectively; 3 biological replicates with technical triplicates in **A** (right); 4 biological replicates for **M**). Statistical tests were calculated by *t*-test (**A**–**C**, **E**, **F**, **I**, **J**, **M**). ns not significant.

We next sought to further understand the impact of galectin-8 in response to TGF-β in CRC cells with a heterozygous mutation by using DLD1 cells harboring a mutant *TGFBR1* allele (TGFBR1*6A), which was shown to act as a tumor susceptibility allele that stimulates cell growth and cancer development when exposed to TGF-β [24]. In addition, DLD1 cells carry *TGFBR2* mutations, which confer selective advantages [25]. *LGALS8* knockdown by shRNA or multiple siRNAs reduced cell adhesion and increased migratory activity in DLD1 cells (Fig. 2A, B; Supplementary Fig. S2A). DLD1 cells produced TGF-β (Supplementary Fig. S2B). Notably, the migratory activity in galectin-8 silencing cells was more prominent by treatment with TGF-β (Supplementary Fig. S2C). The invasiveness of DLD1 cells was also increased after the knockdown of *LGALS8* (Fig. 2C). As in HT29 cells, galectin-8 silencing by shRNA or multiple siRNAs altered the expression of EMT markers (Fig. 2D and Supplementary Fig. S2D) and enhanced the TGF-β-mediated cell migration (Fig. 2E, F), which can be attenuated by the treatment with RAS-I or SRC-I (Fig. 2E, F). Co-localization and interaction between galectin-8 and TβRII were also confirmed through PLA and co-IP in DLD1 cells (Fig. 2G, H). In support of our hypothesis mentioned above, LY2109761, RAS-I, and/or SRC-I diminished the increased migration after the knockdown of *LGALS8* using shRNA or multiple siRNAs in DLD1 cells (Fig. 2I, J). Similarly, RAS and Src activation were suppressed by LY2109761 in *LGALS8* knockdown DLD1 cells (Fig. 2K). Furthermore, PLA results demonstrated that in response to TGF-β treatment, TβRI/TβRII association was enhanced in galectin-8 knockdown DLD1 cells (Fig. 2L, M). Consisently, in parental DLD1 cells, treatment with TGF-β reduced the interaction between endogenous galectin-8 and TβRII (Supplementary Fig. S2E, F). Combining the results from HT29 and DLD1 cells, we showed that galectin-8 regulates TGF-β-mediated noncanonical signaling by disrupting TGF-β binding to TβRII in CRC cells without or with heterozygous *TGFBR1/2* mutations, and represses cell migration.

### Induction of galectin-8 expression in mice transplanted with CRC cells reduces the spontaneous formation of liver metastases in vivo

**Fig. 3:**
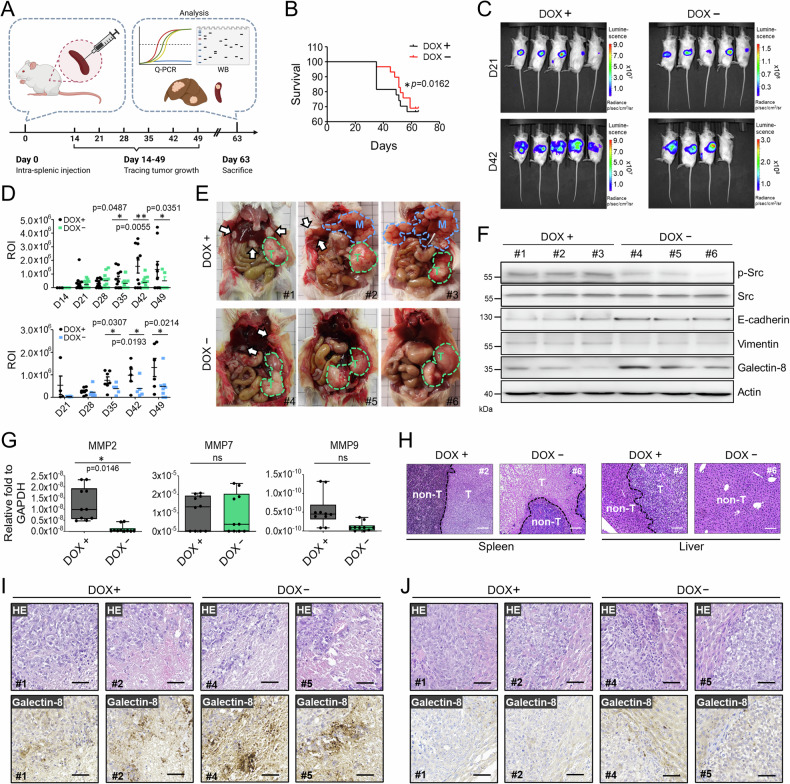
Induction of galectin-8 suppresses liver metastases from intra-splenically implanted CRC cells. **A** Scheme of intra-splenic implantation of galectin-8 Tet-off DLD1-Luc cells and subsequent examination of metastasis. Implanted NOD-SCID mice were treated with or without doxycycline (DOX, 200 µM in drinking water), monitored for tumor burden, or sacrificed for various analyses at indicated days. **B** Kaplan–Meier survival curves of NOD-SCID mice implanted intra-splenically with galectin-8 Tet-off DLD1-Luc cells with or without DOX treatment (200 µM). n = 10 per group. Statistical significance was determined using the log-rank test. **C** Representative bioluminescence images of NOD-SCID mice treated with or without DOX at 21 days and 42 days after intra-splenic implantation of galectin-8 Tet-off DLD1-Luc cells. **D** The bioluminescence intensity of the regions of interest (ROI) detected from primary tumors (upper panel) and metastatic nodules (lower panel) at indicated days after implantation. **E** Gross images of galectin-8 Tet-off DLD1-Luc tumor-bearing mice treated with or without DOX show the primary tumors (green dotted circles, marked as T) and metastatic nodules (arrows and blue dotted circles, marked as M) at day 63 after implantation. **F** Western blot analysis of the expression of phosphorylated and total Src, E-cadherin, Vimentin, and galectin-8 in the primary tumor mass of spleen from galectin-8 Tet-off DLD1-Luc tumor-bearing mice with or without DOX treatment. Actin was used as the loading control. **G** RT-qPCR analysis of mRNA levels of MMP-2, MMP-7, and MMP9 in tumor tissues from the spleen in (**E**). **H** Hematoxylin-eosin (HE) staining showing the boundary between tumorous (T) and non-tumorous (non-T) tissues in the liver and spleen of galectin-8 Tet-off DLD1-Luc tumor-bearing mice. Scale bar = 100 μm. (I and J) HE staining and IHC staining of galectin-8 expression with sections derived from the primary lesions in spleen (**I**) and metastatic lesions in liver (**J**) in (**E**). Scale bar = 50 μm. Results in **D** and **G** are shown as mean ± SD (3 independent experiments in the upper panel of **D**; 3 independent experiments in the lower panel of **D**; 5 biological replicates with technical duplicate for G). Statistical tests in **D** and **G** were calculated by *t*-test. ns not significant.

Metastasis in CRC is a leading cause of death, primarily due to the limited therapies targeting liver metastases [26]. We therefore use DLD1 cells which have high metastatic potential [27] to study the impact of galectin-8 on CRC growth and liver metastasis [27]. We established galectin-8 Tet-off DLD1-Luc cells that constitutively expressed luciferase (Supplementary Fig. S3A) and in which galectin-8 expression could be induced in the absence of doxycycline (DOX) (Supplementary Fig. S3B). Galectin-8 Tet-off DLD1-Luc cells were implanted into the spleens of NOD-SCID mice, and the mice were given drinking water with or without DOX (Fig. 3A). We were able to define the boundary between primary tumors and metastatic nodules by injecting luciferin into the DOX-treated NOD-SCID mice on day 21 after implantation of galectin-8 Tet-off DLD1-Luc cells (Supplementary Fig. S3C). Kaplan–Meier survival curve analysis showed that mice injected with galectin-8 Tet-off DLD1-Luc cells that did not receive DOX (DOX−) had significantly better overall survival than the DOX-treated (DOX+) mice (Fig. 3B). We also observed that the gross bioluminescence of tumors in DOX-untreated mice on day 42 after implantation was generally lower than that of the tumors in DOX-treated mice (Fig. 3C). We measured the intensity of bioluminescence in the region of interest and found that in the absence of DOX, the tumor volumes in the spleen (Fig. 3D, upper panel) and liver (Fig. 3D, lower panel) were significantly reduced over time. Moreover, fewer metastatic nodules were observed in the livers of DOX− than in those of DOX+ mice (Fig. 3E). Lysates of primary tumors isolated from the spleens of DOX-treated or -untreated mice were analyzed by immunoblotting and qRT-PCR. The levels of phosphorylated Src and Vimentin were lower, whereas those of E-cadherin were higher in tumors from DOX− than from DOX+ mice (Fig. 3F). Matrix metalloproteinases (MMPs) are involved throughout CRC pathogenesis, from the initial stages of tumor development to the formation of metastases in secondary organs [28]. The mRNA levels of MMP2, but not MMP7 and MMP9, were significantly reduced in the tumors of DOX− mice (Fig. 3G). Hematoxylin and eosin (HE) staining confirmed the presence of tumor nodules in the livers of the DOX-treated group (Fig. 3H). Additionally, we found that the expression of galectin-8, detected by IHC, in the metastatic nodules of all groups (Fig. 3J) was lower than in the primary tumor tissues in the spleen (Fig. 3I). Together, these results show that the increased expression of galectin-8 in mice transplanted with CRC cells effectively reduced tumor growth and liver metastasis.

### Recombinant galectin-8 inhibits TGF-β receptor activity in CRC cells

**Fig. 4:**
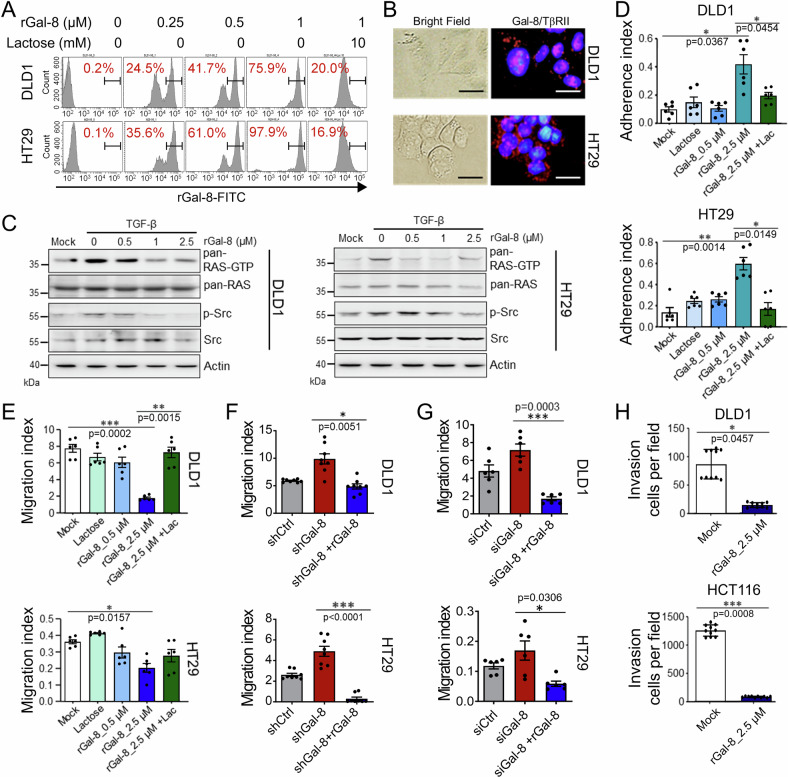
rGal-8 suppresses migration and invasion of CRC cells by targeting TGF-β signaling. **A** FACS shows binding of the indicated amounts of FITC-conjugated rGal-8 with DLD1 and HT29 cells. Lactose (10 mM) blocks the binding of rGal-8 with CRC cells. **B** PLA shows the interaction of galectin-8 (Gal-8) and TβRII on CRC cells. Scale bar = 10 μm. **C** Western blotting shows that rGal-8 affects TGF-β-mediated RAS and Src activities in CRC cells. Cell lysates from untreated DLD1 and HT29 cells (mock) or from cells treated with TGF-β (100 ng/mL) and various amounts of rGal-8 were subjected to immunoblotting with the indicated antibodies. Actin serves as the loading control. Cell adhesion (**D**) and migration (**E**) of DLD1 and HT29 cells treated with the indicated doses of rGal-8 and/or lactose (Lac, 100 mM), analyzed by xCELLigence RTCA. Bar graphs show the average cell indexes at 6 h (**D**) and 48 h (**E**). **F** Cell migration of DLD1 and HT29 cells expressing shCtrl or shGal-8 in the absence or presence of rGal-8 (2.5 µM) analyzed by xCELLigence RTCA at 48 h. **G** Cell migration of DLD1 and HT29 cells treated with siCtrl or siGal-8 in the absence or presence of rGal-8 (2.5 µM) analyzed by xCELLigence RTCA at 48 h. **H** Invasive activities of DLD1 and HCT116 cells treated with rGal-8 for 24 h analyzed by xCELLigence RTCA. Results in **D**, **E**, **F**, **G**, **H** are shown as mean ± SD (3, 3, 4 and 3 biological replicates with technical duplicates for each in **D**, **E**, **F**, **G**, respectively; 2 biological replicates with technical quintuplicates in **H**). Statistical tests were calculated by *t*-test.

Having demonstrated the anti-metastatic role of galectin-8 in vitro and in vivo, we next evaluated whether treatment of CRC cells with recombinant galectin-8 (rGal-8) mimicked the effect of endogenous galectin-8 on CRC cells. First, we observed a dose-dependent increase in FITC-labeled rGal-8 binding to DLD1 and HT29 cells (Fig. 4A). The PLA was also used to verify that treatment with rGal-8 promoted the association between galectin-8 and TβRII on DLD1 and HT29 cells (Fig. 4B). Moreover, the TGF-β-induced activation of RAS and Src was blocked in a dose-dependent manner following the treatment of DLD1 and HT29 cells with rGal-8 (Fig. 4C). Treatment with rGal-8 also enhanced DLD1 and HT29 cell adhesion but suppressed migration, and these effects were reversed by lactose treatment (Fig. 4D, E). Consistent with these results, rGal-8 treatment delayed cell migration in wound healing assays (Supplementary Fig. S4A, B). In contrast, adhesion and migration of HCoEpi cells were not affected by rGal-8 treatment (Supplementary Fig. S4C, D). rGal-8 also did not co-localize with TβRII of HCoEpi cells (Supplementary Fig. S4E), even though rGal-8 was able to bind with HCoEpi cells to a certain extent (Supplementary Fig. S4F) and the HCoEpi cells expressed low level of TβRII (Supplementary Fig. S4G). Moreover, administration of rGal-8 abrogated the increase in migratory activity resulting from knockdown of *LGALS8* by shRNA (Fig. 4F) or multiple siRNAs (Fig. 4G) in DLD1 and HT29 cells, and significantly suppressed the invasive activities of DLD1 and HCT116 cells (Fig. 4H). Thus, in CRC cells, rGal-8 shows similar anti-migratory activity as endogenous galectin-8.

### rGal-8 induces JNK-dependent apoptosis in CRC cells

**Fig. 5:**
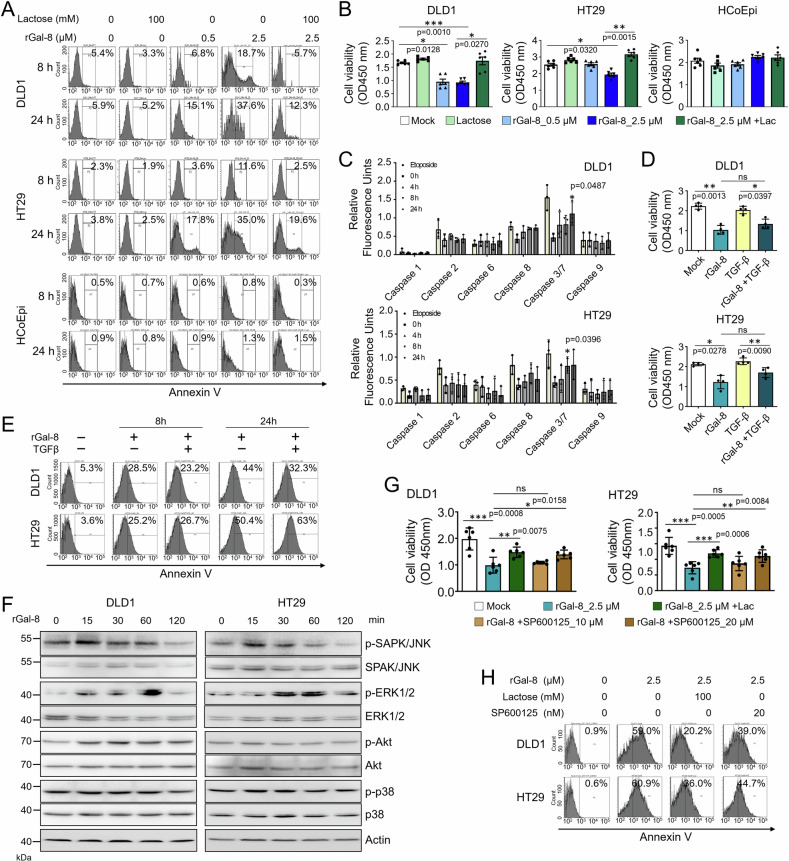
rGal-8 triggers apoptosis in CRC cells by inducing transient JNK activation. The level of cell apoptosis, determined by Annexin V staining (**A**), and cell viability, determined by MTT assay (**B**), of DLD1, HT29, and HCoEpi cells treated with various doses of rGal-8 in the presence or absence of lactose (Lac, 100 mM). **C** The activities of caspases in DLD1 and HT29 cells treated with rGal-8 (2.5 µM) or etoposide (10 µM, positive control of caspase activation) for the indicated time points were analyzed based on the cleavage of each substrate. **D** Cell viability of DLD1 and HT29 cells treated with rGal-8 (2.5 µM) or/and TGF-β (100 ng/mL) for 72 h determined by MTT assay. **E** FACS shows the percentage of Annexin V^+^ apoptotic DLD1 or HT29 cells treated with rGal-8 (2.5 µM) and/or TGF-β (100 ng/mL) for the indicated time points. **F** Western blotting shows the expression level of phosphorylated and total JNK, ERK1/2, AKT and p38 in DLD1 and HT29 cells treated with rGal-8 (2.5 µM) for the indicated time points. Cell viability determined by MTT assay (**G**), and apoptosis determined by Annexin V staining (H) of DLD1 and HT29 cells treated with rGal-8 (2.5 µM) alone or together with JNK inhibitor (SP600125) at the indicated doses for 72 h (**G**) and 24 h (**H**). Lactose (Lac, 100 mM) was used to block the effect of rGal-8 in some groups. Results in **B**, **C**, **D**, **G** are shown as mean ± SD (3 biological replicates with technical duplicates for each in **B**, **C**, **G**; two biological replicates with technical duplicates for **D**). Statistical tests were calculated by *t*-test.

We then explored the impact of rGal-8 on the viability of CRC cells as a previous study demonstrated the pro-apoptotic effects of galectin-8 in H1299 lung carcinoma cells [29]. We found that while rGal-8 did not affect the viability of normal HCoEpi cells, it elicited apoptosis in DLD1 and HT29 cells in a dose- and time-dependent manner (Fig. 5A), and reduced CRC cell viability (Fig. 5B). The effects of rGal-8 on the cell death of CRC cells can be prevented by lactose (Fig. 5A, B). Furthermore, rGal-8 treatment induced the activation of caspase 3 and caspase 7, the key effectors of apoptosis (Fig. 5C), but did not influence cell cycle distribution in DLD1 and HT29 cells (Supplementary Fig. S5A). It is noted that the cell viability reduction and apoptosis resulting from rGal-8 treatment could not be rescued by TGF-β administration (Fig. 5D, E). Consistently, treatment with LY2109761 did not reduce rGal-8-mediated apoptosis (Supplementary Fig. S5B). We thus turned into the alternative pathways and found that rGal-8 elicited temporal activation of JNK and ERK in CRC cells, which can not be affected by LY2109761 treatment (Fig. 5F and Supplementary Fig. S5C). The activation of JNK appeared to be critical for the pro-apoptotic effect of rGal-8 on CRC cells, as the JNK inhibitor SP600125, but not the ERK inhibitor PD98059, attenuated the apoptosis and the loss of viability caused by rGal-8 (Fig. 5G, H; Supplementary Fig. S5D). Together, although knockdown of galctin-8 did not change the survival of CRC cells, rGal-8 treatment triggers JNK-dependent apoptosis in CRC cells, but not normal HCoEpi cells.

### Knockdown of *B4GALT1* abolishes the galectin-8-mediated anti-metastatic effects

**Fig. 6:**
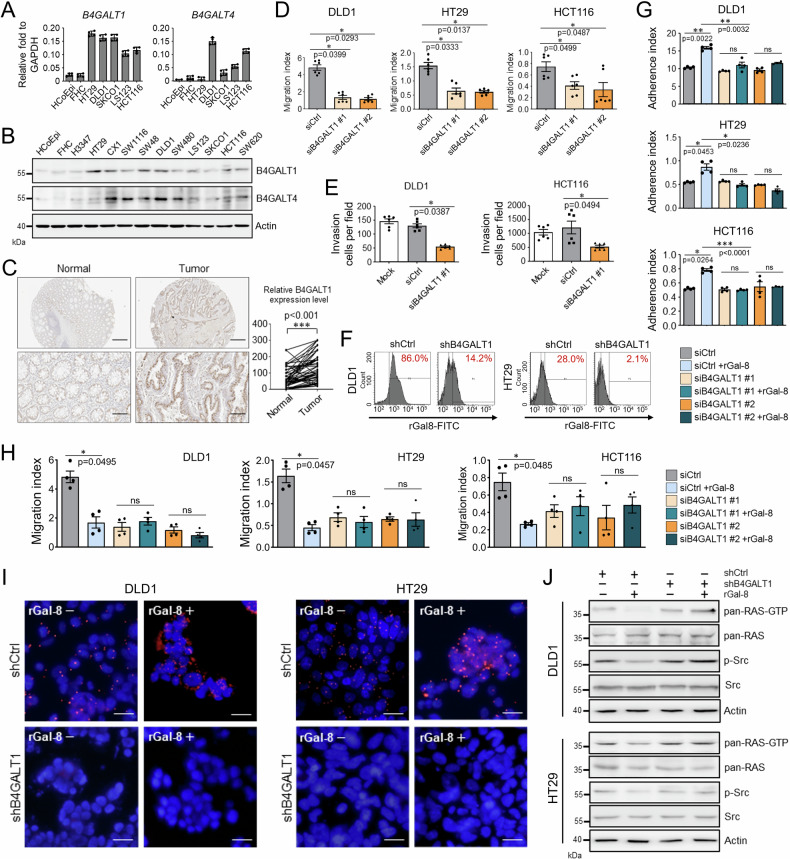
B4GALT1 supports the migratory activity of CRC cells and is required for the anti-metastatic effect of galectin-8. **A** RT-qPCR shows the relative B4GALT1 and B4GALT4 mRNA levels normalized by GAPDH mRNA levels in non-tumorous cells (HCoEpi and FHC) and various CRC lines. **B** Western blotting shows protein expression of B4GALT1 and B4GALT4 in non-tumorous cells (HCoEpi and FHC) and in different CRC lines. Actin was used as a protein loading control. **C** Immunohistochemical staining of B4GALT1 in paired non-tumor and tumor tissues from CRC patients (left). The right panel shows the distribution of immunoactivity scores of B4GALT1 expression in normal and primary CRC tissues (n = 57). Scores were determined as the product of staining intensity and percentage of positive cells. Scale bar = 200 μm (top); 50 μm (bottom). Cell migration (**D**) and invasive activities (**E**) of the indicated CRC cells treated with siCtrl or siB4GALT1 (#1 and #2 in **D**, #1 in **E**) were analyzed at endpoint (**D**, 48 h; **E**, 24 h) using xCELLigence RTCA. **F** FACS shows the percentage of cells binding with FITC-conjugated rGal-8 among DLD1 and HT29 cells expressing shCtrl or shB4GALT1. Cell adhesion (**G**) and migration (**H**) activities of DLD1, HT29, and (Fig. 5D, E) cells treated with siCtrl or siB4GALT1 (#1 or #2) (25 pmol/10^6^ cells) in the presence or absence of rGal-8 (2.5 µM). Results were analyzed by xCELLigence RTCA at 6 h (**E**) and 48 h (**F**) after treatment. **I** PLA shows the effects of knockdown of *B4GALT1* on the interaction between TβRII and galectin-8 in DLD1 and HT29 cells with (rGal-8+) or without (rGal-8−) rGal-8 treatment. DAPI staining was used to label cell nuclei. Scale bar = 10 µm. **J** Western blotting shows the expression of activated, phosphorylated, and total proteins of RAS or Src in DLD1 and HT29 cells treated as indicated. rGal-8 at 2.5 µM was used. Actin was used as the loading control. Results in **A**, **D**, **E**, **G**, **H** are shown as mean ± SD (2 biological replicates with technical duplicates for each in **A**, **G**, **H**; 3 biological replicates with technical duplicates for **D**, **E**). Statistical tests were calculated by *t*-test. ns not significant.

We next sought to determine which of the genes involved in the synthesis of N-glycan moieties contribute to the effects mediated by galectin-8. We evaluated the expression of genes that are most frequently altered in malignant cancers [30] (Supplementary Fig. S6A) and found that the mRNA and protein levels of B4GALT1 and B4GALT4 were increased in CRC cells (Fig. 6A, B). The increase in protein levels of B4GALT1 and B4GALT4 was consistent with the previously reported malignant status of CRC cell lines [21] (Fig. 6B). The expression of B4GALT1 and B4GALT4 in paired normal and tumor colon tissues was further examined by IHC. In contrast to B4GALT4 expression (Supplementary Fig. S6B), a significant increase in *B4GALT1* expression was observed in CRC tissues compared with normal colon samples (Fig. 6C).

Knockdown of *B4GALT1* by shRNA or siRNA (Supplementary Fig. S6C) in DLD1, HT29, and HCT116 CRC cells did not affect their adhesion (Supplementary Fig. S6D, E) but significantly attenuated their migration (Fig. 6D and Supplementary Fig. S6F) and invasion (Fig. 6E and Supplementary Fig. S6G); of note, cell viability was not affected (Supplementary Fig. S6H). Taken together, these results suggest that B4GALT1 attributed to the malignant properties of CRC cells.

We next investigated whether the anti-metastatic effect of galectin-8 was dependent on the recognition of glycans synthesized by B4GALT1. A synthetic disaccharide compound, 6SI-OMe (Supplementary Fig. S7A), which preferentially blocks the interaction between glycans and the CRD of galectin-8 [31], was used for this purpose. The presence of 6SI-OMe prevented the binding of FITC-labeled rGal-8 to DLD1 cells (Supplementary Fig. S7B). In addition, we found that 6SI-OMe significantly abolished the effect of rGal-8 (i.e., promoting cell adhesion and inhibiting the migratory activity of DLD1 cells) in a dose-dependent manner (Supplementary Fig. S7C, D). This indicates that the mode of action of rGal-8 in CRC cells was dependent on carbohydrate recognition.

We next examined whether the depletion of B4GALT1 affected the activity of rGal-8 in CRC cells. Remarkably, the binding of rGal-8 to DLD1 and HT29 cells was largely abolished following B4GALT1 depletion (Fig. 6F). Moreover, knockdown of *B4GALT1* with shB4GALT1 or siB4GALT1 reduced the effects of rGal-8 on cell adhesion (Fig. 6G and Supplementary Fig. S7E) and migration (Fig. 6H and Supplementary Fig. S7F), regardless of the presence of TGF-β. The interaction between galectin-8 and TβRII in DLD1 and HT29 cells was abolished after the knockdown of *B4GALT1*, either in the presence or absence of rGal-8 (Fig. 6I). Concordantly, the suppressive effect of rGal-8 on RAS activation and Src phosphorylation was diminished in *B4GALT1*-silenced CRC cells (Fig. 6J). These results suggest that both endogenous galectin-8 and rGal-8 exerted anti-metastatic activities via a similar mechanism, which was dependent on the expression of *B4GALT1* in CRC cells.

## Discussion

**Fig. 7:**
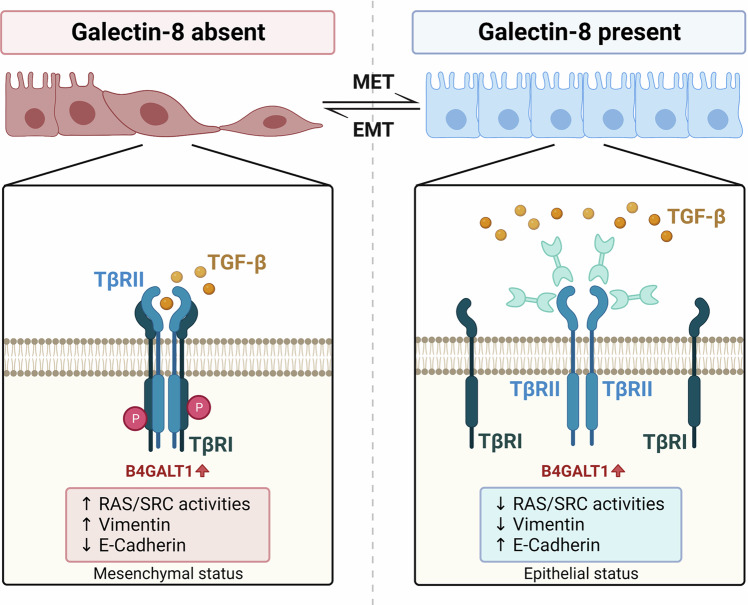
Proposed model of the action of galectin-8 in CRC. CRC cells showed increased levels of B4GALT1. In the absence of galectin-8 (left), TGF-β binds to TβRII, thereby promoting EMT. In the presence of galectin-8 (right), galectin-8 binds to TβRII through B4GALT1-mediated galactosylation of N-glycans, resulting in a decrease of TGF-β signaling-mediated EMT.

In this study, we showed that galectin-8 disrupted the initiation response to TGF-β by impeding the assembly of the TβRI/TβRII receptor complex, thereby preventing metastasis (Fig. 7). We also found that the B4GALT1 was required for the galectin-8-mediated modulation of CRC progression. Our findings highlight the association between CRC progression, glycome dysregulation, and carbohydrate recognition proteins. A previous study also showed that altered expression of a glycan synthesis enzyme in metastatic melanoma resulted in a shift of the glycome that favored galectin-8 binding [32]. The mechanism by which galectin-8 is upregulated in less aggressive CRC cells and downregulation in more malignant cells remains unclear. However, its expression in human cancer cells appears to be related to tumor stages [33, 34]. Consistnetly, our results showed that galectin-8 expression is downregulated during CRC progression. The expression of galectin-8 is significantly lower in T4 stage than that in T1 stage. In the T classification, T1 represents an invasion of the submucosa in CRC, whereas T4 indicates tumor extension through all layers of the colon and an invasion of visceral peritoneum or adjacent structures [35]. Depletion of galectin-8 promotes the migration of CRC cells, which can be rescued by the addition of rGal-8. In addition to being anti-metastatic, rGal-8 can induce apoptosis of CRC cells via JNK signaling. Whether the induction of apoptosis mediated by galectin-8 depends on B4GALT1 should be further examined. On the other hand, although we demonstrated the extracellular effect of galectin-8, we cannot exclude its potential intracellular role in CRC cells, as galectin-8 has been shown to support autophagy [36].

In addition to being produced by CRC cells, galectin-8 is also expressed by immune cells such as plasma cells and regulatory T cells, whereby modulating cell differentiation [31, 37]. Therefore, galectin-8 produced by non-CRC cells may also contribute to the regulation of tumor progression in vivo. The anti-malignant effects of galectin-8 in the tumor microenvironment might also be related to its anti-inflammatory properties and its regulation of T cell homeostasis [37, 38]. Because inflammation increases the risk of developing many types of cancer, the anti-inflammatory properties of galectin-8 could contribute to the anti-malignant effect observed in vivo.

The role of B4GALT1 in cancer remains controversial. The *B4GALT1* promoter is more heavily methylated in invasive CRC lesions than in non-invasive CRC lesions [39], and B4GALT1 expression has been suggested as a prognostic biomarker in renal, bladder, and lung cancer [40–42]. Here, we demonstrated a positive correlation between increased B4GALT1 expression and cancerous CRC lesions, suggesting that B4GALT1 is a potential biomarker in CRC progression. At the same time, galectin-8 could then suppress cell migratory activity of B4GALT1-expressing CRC cells to reduce tumor progression. In addition to CRC, TGF-β signaling has been shown to promote the development and aggressiveness of several types of cancers, including gastric, lung, breast, and liver cancers [43–46]. We also found that breast, gastric, liver, and lung cancer cell lines expressed both galectin-8 and B4GALT1 (Supplementary Fig. S8A, B). Moreover, the migratory activities of these B4GALT1-expressing cancer cells were suppressed by rGal-8, as observed in the CRC cell line experiments (Supplementary Fig. S8C). These results suggest that the B4GALT1-dependent anti-metastatic effect of galectin-8 occurs in different types of cancer.

Previous studies have shown that galectins retain the glycoproteins at the cell surface by reducing receptor endocytosis [47]. TβRI binding to the TβRII and TGF-β complex necessitates the interaction between TβRII and TGF-β for TβRI recruitment [48]. We found that rGal-8 treatment in CRC cells reduces TGF-β-mediated signaling by attenuating TGF-β binding to TβRII. Flow cytometric analysis showed that cell surface TβRII levels were transiently increased and then slightly decreased 2 h after TGF-β stimulation; this was independent of endogenous galectin-8 concentration (Supplementary Fig. S9A, B). Additionally, rGal-8 maintained stable surface expression of TβRII, counteracting the typical transient increase and subsequent decrease observed after TGF-β stimulation (Supplementary Fig. S9C, D), indicating a multifaceted impact on TGF-β-mediated responses by galectin-8 in CRC cells.

In conclusion, we demonstrated the anti-metastatic and apoptosis-inducing effects of galectin-8 in CRC. Thus, modulating the expression of a specific glycan-recognition protein, such as through the targeted delivery of rGal-8, may represent another therapeutic strategy in CRC and other cancers.

## Supplementary information

Supplementary inforamtion

Uncropped westernblot images

## Data Availability

All data generated or analyzed during this study are available from the corresponding author on reasonable request.
