# Supplementary material for: B4GALT1-dependent galectin-8 binding with TGF-β receptor suppresses colorectal cancer progression and metastasis
Source: Cell Death Dis. 2024 Sep 4;15(9):654. doi: 10.1038/s41419-024-07028-3 (PMC11375092; doi:10.1038/s41419-024-07028-3)
Supplement: Supplementary file 1 — Supplementary inforamtion [file 41419_2024_7028_MOESM1_ESM.pdf]

Supplementary information

B4GALT1-dependent galectin-8 binding with TGF-β receptor suppresses colorectal cancer progression and metastasis.

Tzu-Hui Hsu[1], Yu-Chan Chang[1,2], Yi-Yuan Lee[1], Chi-Long Chen[3, 4], Michael Hsiao[1], Fan-Ru Lin[1], Li-Han Chen[5,6], Chun-Hung Lin[5,6], Takashi Angata[5], Fu-Tong Liu[7] and Kuo-I Lin[1*]

Affiliations:

[1]Genomics Research Center, Academia Sinica, Taipei 115, Taiwan

[2]Department of Biomedical Imaging and Radiological Sciences, National Yang Ming Chiao Tung University, Taipei 112, Taiwan

[3]Department of Pathology, Taipei Medical University Hospital, Taipei 110, Taiwan

[4]Department of Pathology, College of Medicine, Taipei Medical University, Taipei 110, Taiwan

[5]Institute of Biological Chemistry, Academia Sinica, Taipei 115, Taiwan

[6]Department of Chemistry, National Taiwan University, Taipei, 106, Taiwan

[7]Institute of Biomedical Sciences, Academia Sinica, Taipei 115, Taiwan

*Correspondence: kuoilin@gate.sinica.edu.tw

Correspondence:

Kuo-I Lin, Genomics Research Center, Academia Sinica, No. 128, Section 2, Academia Road, Nangang District, Taipei 115, Taiwan. Phone: +886-2-2787-1253, Fax: +886-2-2789-9931; email: kuoilin@gate.sinica.edu.tw

Table of contents

- Supplementary Materials and Methods
- Supplementary References
- Supplementary Table
- Supplementary Figures and Legends

Supplementary Table 1. The sequences of primers for qPCR

Supplementary Figure 1. Expression of *LGALS8* in CRC cells and determination of knockdown efficiency of shRNAs and siRNAs targeting *LGALS8*.

Supplementary Figure 2. Knockdown of *LGALS8* promotes TGF-β response in DLD1 cells.

Supplementary Figure 3. Induction of intrasplenic tumors and liver metastases by implantation of luciferase-expressing galectin-8 Tet-off DLD1 cells in NOD-SCID mice.

Supplementary Figure 4. The effect of rGal-8 on CRC cells and normal human intestinal epithelial cells.

Supplementary Figure 5. The effect of rGal-8 on the cell cycle and apoptosis of CRC cells.

Supplementary Figure 6. Knockdown of *B4GALT1* reduces invasive and migratory activities of CRC cells but does not affect cell adhesion and viability.

Supplementary Figure 7. Knockdown of *B4GALT1* abrogates the anti-metastatic effects mediated by rGal-8.

Supplementary Figure 8. The anti-metastatic effects of rGal-8 in other types of cancer cells.

Supplementary Figure 9. Cell surface expression of TβRII in the presence or absence of galectin-8 and TGF-β.

Supplementary Materials and Methods

Chemicals, reagents, and recombinant galectin-8 protein preparation

In some experiments, recombinant human TGF-β (Cat. No. 100-21, PeproTech, NJ, USA, 100 mg/mL in stock), etoposide (Cat. No. 33419-42-0, Sigma-Aldrich, MO, USA, 10 mM in stock), LY2109761 (Cat. No. 15409, Cayman chemical, MI, USA, 10 mM in stock), pan-RAS inhibitor lonafarnib (Cat. No. SML1457, Sigma-Aldrich, MA, USA, 100 mM in stock), SRC inhibitor dasatinib (Cat. No. SML2589, Sigma-Aldrich, 30 mM in stock), JNK inhibitor SP600125 (Cat. No. tlrl-sp60, InvivoGen, 50 mM in stock) or ERK inhibitor PD98059 (Cat. No. tlrl-pd98, InvivoGen, CA, USA, 100 mM in stock) was used to treat CRC cells. 0.01% DMSO (Sigma-Aldrich) treatment was used as the control. The compound 6SI-OMe was synthesized by Dr. Chun-Hung Lin's laboratory (Academia Sinica) as previously described (ref. 1). To produce rGal-8, the complete cDNA of human *LGALS8* (948 bp) was amplified by PCR with specific primers containing NdeI and BamHI restriction enzyme sites. The amplified *LGALS8* full-length cDNA fragment was digested with the restriction enzymes and inserted into the plasmid pET-15b (Cat. No. 69661, Sigma-Aldrich) carrying an N-terminal His-Tag. The recombinant plasmid was transformed to BL21 (DE3) bacteria and the sequence of full-length *LGALS8* was verified. rGal-8 protein was expressed by induction with 0.4 mM isopropyl-β-D-thiogalactopyranoside (Cat. No. IPT001, Bioman Scientific, Taipei, Taiwan) for 4 h at 37°C in LB medium containing 100 μg/mL ampicillin. Cell culture was centrifuged at 6,000 *g* for 20 min and re-suspended in PBS. The cell suspension was then disrupted by homogenization at 900 bar twice and the lysate was clarified by centrifugation at 20,000 *g* for 30 min, followed by filtration with 0.2 μm filters. Clarified lysate was loaded into a packed β-lactose Sepharose 6B column (Cat. No. 9012-36-6, Sigma-Aldrich) and washed with PBS. The bound proteins were eluted with PBS containing ~0–0.1M β-lactose. The collected fractions were analyzed with 12% SDS-

PAGE and loaded onto a packed Q Sepharose FF column (Cat. No. 17-0510-01, GE Healthcare, IL, USA) for further purification and endotoxin removal. rGal-8, with a concentration at 5 mg/mL, purity at 95.8%, and endotoxin content below 2 EU/mg protein, was obtained.

Patients

Patients who received preoperative chemotherapy or radiation therapy or who received incomplete surgical resection were excluded. All cases were staged according to the Cancer Staging System of the American Joint Committee on Cancer (AJCC), and the histological cancer type was classified according to the World Health Organization classification. All tissue sections with tumor lesions and non-tumor parts were fixed by 10% formalin, dehydrated and paraffin embedded. Sections were histologically examined by IHC stain.

Cell lines

Cell lines, including MDAMB231, MCF7, T47D, TMK1, SCM1, Hep3B, and H1299 cells, were purchased from the American Type Culture Collection (ATCC). All cell lines were grown in RPMI1640 (Cat. No. 11875, Gibco, Thermo Fisher Scientific, MA, USA), supplemented with 10% FBS (Cat. No. 26140079, Gibco) and penicillin (100 U/mL)/streptomycin (100 μg/mL) (Cat. No. 15140148, Gibco). Cells were incubated at 37°C under an atmosphere containing 5% CO_2 and tested for mycoplasma contamination using EZ-PCR™ Mycoplasma Detection Kit (Cat. No. SKU: 20-700-20, Sartorius).

Total RNA isolation and RT-qPCR

Real-time qPCR analysis was performed on an Applied Biosystems™ ABI 7500 Fast

Real-Time PCR System (Thermo Fisher Scientific) using Power SYBR Green Master Mix (Cat. No. 4367659, Thermo Fisher Scientific) starting with 1 μg RNA isolated by TOOLSmart RNA Extractor (Cat. No. DPT-BD24, Tools, Taipei, Taiwan), according to our previous publication (ref. 2). Tumor tissues were frozen in liquid nitrogen and homogenized with a mortar prior to RNA isolation. 1 μg RNA was used for cDNA synthesis. The qPCR primer sequences used in this study are provided in Supplemental Table 1. Primers were designed following the instructions at the OriGene website. The Ct values for each gene were normalized to the Ct values of GAPDH mRNA using the ΔCt equation. Relative fold of mRNA levels was calculated by ΔΔCt.

Antibodies used in immunoblotting

Antibodies used in the immunoblotting are rabbit anti-galectin-8 (Cat. No. ab69631, Abcam, Cambridge, UK), rabbit anti-B4GALT1 (Cat. No. ab121326, Abcam), mouse anti-B4GALT4 (Cat. No. ab169604, Abcam), mouse anti-TβRII (Cat. No. ab78419, Abcam), rabbit anti-E-cadherin (Cat. No. GTX100443, GenTex, CA, USA), rabbit anti-Vimentin (Cat. No. GTX100619, GenTex), rabbit anti-SLUG (Cat. No. GTX128796, GeneTex), rabbit anti-TWIST (Cat. No. GTX127310, GeneTex), goat anti-SNAIL (Cat. No. ab53519, Abcam), rabbit anti-Lamin A (Cat. No. sc-20680, Santa Cruz), mouse anti-α-tubulin (Cat. No. 62204, Thermo Fisher Scientific), mouse anti-Src (Cat. No. GTX19336, GeneTex), rabbit anti-Src phospho-Y416 (Cat. No. GTX81151, GeneTex), rabbit anti-SAPK/JNK (Cat. No. 9252, Cell Signaling), rabbit anti-SAPK/JNK at phosphor-T183/Y185 (Cat. No. 9251, Cell Signaling), rabbit anti-ERK (Cat. No. 4695, Cell Signaling), rabbit anti-ERK at phosphor-T202/Y204 (Cat. No. 4370, Cell Signaling), rabbit anti-Akt (Cat. No. 9272, Cell Signaling), rabbit anti-Akt at phospho-S473 (Cat. No. 4058, Cell Signaling), rabbit anti-p38 (Cat. No. 9212, Cell Signaling) and rabbit anti-p38 at phosphor-T180/Y182 (Cat. No. 9211, Cell Signaling), and mouse anti-actin

(Cat. No. MAB1501, Merck Millipore, MA, USA). The primary antibodies were prepared in 5% skim milk solution in PBST solution at a dilution of 1:1000.

Immunoblotting

Protein lysates were prepared by resuspending pelleted cells in a lysis buffer containing 125 mM Tri-HCl (pH 6.8), 1% w/v SDS, 20 mM dithiothreitol, 1 mM phenylmethylsulfonylfluoride, leupeptin (4 mg/mL), aprotinin (4 mg/mL), pepstatin (1 mg/mL) (all from Sigma-Aldrich), protease and phosphatase inhibitors (Cat. No. 78440, Thermo Fisher Scientific, 100x in stock) for 30 min at 4°C. Sample preparation and immunoblotting procedures were performed as previously described (ref. 2). In some cases, whole tissue samples from mice were collected immediately after sacrificing mice, and placed in liquid nitrogen. Frozen tissue samples were ground into fine powder on ice and homogenized in 300 μL lysis buffer. Samples were then solubilized by sonication with a Q700 sonicator (Qsonica, Taipei, Taiwan) on ice for 5 min and centrifuged at 13,200 x g for 30 min at 4°C. Nuclear extracts were prepared by NE-PER™ Nuclear and Cytoplasmic Extraction Reagents (Cat. No. 78833, Thermo Fisher Scientific) following the manufacturer's instructions. Equal amounts of protein (30–60 μg) in the SDS sample loading buffer were separated by 12% SDS-PAGE and transferred to Immobilon-PSQ PVDF membranes (Millipore, Milan, Italy). Membranes were blocked in PBS-dissolved 5% non-fat dry milk and then immunostained with appropriate primary Ab (diluted 1:1000) followed by incubating with the corresponding HRP-conjugated secondary antibody (diluted 1:5000). Immunoreactive complexes were visualized with ImageQuant LAS-4000 (GE Healthcare, IL, USA).

Lentiviral transduction and siRNA transfection

VSV-G-pseudotyped lentivirus was purchased from the RNAi core facility, Academia

Sinica, Taipei, Taiwan. A lentiviral vector carrying scramble shRNA (5'-CCGGCCTAAGGTTAAGTCGCCCTCGCTCGAGCGAGGGCGACTTAACCTTAGGTTTTT-3', ASN0000000004) was used as control. shRNA sequences targeting B4GALT1 are: shB4GALT1#1: 5'-CCGGGCTCTAAGTAAACAACAGTTTCTCGAGAAACTGTTGTTTACTTAGAGCTTTTTG-3' (TRCN0000034839) and shB4GALT1#2: 5'-CCGGGTGGCAAAGCAGAACCCAAATCTCGAGATTTGGGTTCTGCTTTGCCACTTTTTG-3' (TRCN0000034843). shRNA sequences targeting human Galectin-8 are shGal-8#1: 5'-CCGGCCTGGAACTTTGATTGTGATACTCGAGTATCACAATCAAAGTTCCAGGTTTTTG-3' (TRCN0000057354) and shGal-8#2: 5'-CCGGGCAAAGTGAATATTCACTCAACTCGAGTTGAGTGAATATTCACTTTGCTTTTTG-3' (TRCN0000057355). pLKO-CMV-luciferase-puro, which carries luciferase cDNA driven by the pCMV promoter on the pLKO lentiviral vector, was provided by Dr. M. Hsiao (Genomics Research Center, Academia Sinica, Taiwan). The Lenti-X Tet-Off Advanced Inducible Expression System (Cat. No. 632163, Clontech Laboratories, CA, USA) was used to construct a pTRE-galectin-8 vector according to the manufacturer's instructions. Human *LGALS8* cDNA (BC015818.1) was cloned into the pTRE-Tight plasmid (Cat. No. 631059, Clontech Laboratories) between NdeI and BamHI sites. Lentiviral vector preparation and transduction were performed as described previously (ref. 3). Briefly, CRC cells were plated in 10-cm plates at a cell density of 1×10^7/well in 4 mL culture medium. Twenty-four hours later, cells were transduced overnight with the appropriate lentiviral vector at an MOI of 1–4 in the presence of polybrene (5 µg/mL; Cat. No. H9268, Sigma-Aldrich). Cells were then washed with phosphate-buffered saline (PBS) and cultured for 24 hours. DLD-1 cells were first transduced with a lentiviral vector containing the modified firefly (*Photinus pyralis*) luciferase to produce

DLD-1-Luc cells, followed by transduction with the Gal-8-Tet-off lentiviral vector. Galectin-8 Tet-off DLD1-Luc stably transduced cells were established by selection with puromycin (Cat. No. P8833, Sigma-Aldrich, 1 μg/mL) for at least 14 days. In one experiment, luciferase activity of galectin-8-Tet-off DLD1-Luc cells was measured using the Victor3 1420 Multilabel Counter (PerkinElmer, MA, USA) with a Dual-Luciferase Reporter Assay System (Cat. No. E1910, Promega, WI, USA). In some experiments, siRNA was used to knock down the expression of B4GALT1 or galectin-8 by the TriFECTa RNAi kit (Integrated DNA Technologies, IA, USA). siRNAs were transfected into CRC cells using Lipofectamine RNAiMAX transfection reagent (Cat. No. 13778100, Thermo Fisher Scientific, MA, USA) as described previously (ref. 4). The siRNA sequences and reference numbers are: siB4GALT1#1: 5'-GGCAUGCUAUGUAAACUUGAAUUTC-3' (Ref: 101701209), siB4GALT1#2: 5'-GUCUCUGCUCUAAGUAAACAACAGT-3' (Ref: 101701212), siB4GALT1#3: 5'-CAUUUCCGUUGCAAUGGAUAAGUTT-3' (Ref: 101701215), hs.Ri.LGALS8.13.1: 5'-GCUUUACCAAGUAAUUGGCAUGACA-3' (Ref: 105705703), hs.Ri.LGALS8.13.2: 5'-GUCCUUAAACAACCUACAGAAUATC-3' (Ref: 105705706) and hs.Ri.LGALS8.13.3: 5'-CGCCUGAAUAUUAAAGCAUUUGUAA-3' (Ref: 105705709) All sequences used in the knockdown experiments recognize distinct regions of the major transcript variants of their target genes.

Measurement of cell adherence, migration and invasion

Cell adherence was monitored with E-plates (Cat. No. 5232368001, ACEA Biosciences) using the xCELLigence RTCA MP instrument (ACEA Biosciences). Cell migration activity was monitored in real-time with CIM-plates (Cat. No. 5665817001, ACEA Biosciences, CA, USA) by the xCELLigence RTCA DP instrument (ACEA Biosciences). Cell invasion activity was evaluated using a Boyden chamber (Neuro Probe) with an

8-μm pore (GE Healthcare). In details, for cell adherence analysis, a total of 5×10^5 cells were seeded in each well. After five washes with PBS, the impedance value of each well was automatically recorded by the MP instrument after 6 hours of incubation. Values for cell adherence were normalized to the cell index at the original stage. For cell migration activity analysis, cells (1×10^5) were seeded in the upper chamber in wells of CIM-plates in 2% FBS-containing media. The lower chambers were filled with a complete medium containing 20% FBS as a chemoattractant. The impedance value of each well was automatically recorded by the DP instrument for 48 hours and expressed as the migration index value. For cell invasion assay, polycarbonate filters were pre-coated with 10 mg/ml of Matrigel (BD Biosciences) on the upper side. Medium containing 10% serum was added to the lower compartment of the chamber. Cells resuspended in serum-free medium were seeded (1.5×10^4/well) in the upper compartment. Twenty-four hours later, cells were stained with Giemsa solution (Merck), and five random fields were counted in each well.

Enzyme-linked immunosorbent assay (ELISA)

A human TGF beta 1 ELISA kit (Cat. No. ab100647, Abcam) was used to measure the levels of TGF-β in the culture supernatant, and a human galectin-8 ELISA kit (Cat. No. MBS722330, MyBioSource, CA, USA) was used to measure the levels of galectin-8 in the culture supernatant, according to the instructions provided by the manufacturer. Fresh samples were concentrated by Vivaspin Turbo 15 (Cat: VS15T21, Sartorius Stedim Biotech, French). ELISA was performed in triplicate, and the OD was read by a 96-well Spectra Max M2 microplate reader.

Proximity ligation assay (PLA)

PLA was performed using the Duolink in Situ Red Starter Kit (Cat. No. DUO92101,

Sigma-Aldrich), according to the manufacturer's instructions. Briefly, CRC cells were seeded on a chamber slide (Millipore). Forty-eight hours later, cells were fixed with 4% paraformaldehyde, permeabilized with 0.05% Triton and washed with washing buffer A (Cat. No. DUO82046-1EA, Sigma-Aldrich). Cells were then blocked with blocking solution (Cat. No. DUO82007, Sigma-Aldrich) at 4°C for 30 min and immunolabeled with primary antibody pairs, including rabbit anti-galectin-8 (1:200, Cat. No. ab69631, Abcam)/mouse anti-TβRII (1:200, Cat. No. ab78419, Abcam) and goat anti-TβRI (1:200, Cat. No. sc-33933, Santa Cruz)/mouse anti-TβRII (1:200) at 4°C overnight. Cells were then washed with washing buffer B (Cat. No. DUO8204-1EA, Sigma-Aldrich) and incubated with each PLA probe (anti-mouse PLUS and PLA probe anti-rabbit MINUS) at 4°C for 60 min. The probes were ligated with the ligation solution and amplified using the amplification solution. Coverslips were mounted on glass slides using Duolink *in situ* mounting medium containing DAPI (Sigma Aldrich). PLA images were acquired using an Olympus IX71 camera (Olympus Life Science, Tokyo, Japan) with excitation at 360 nm and emission at 461 nm for DAPI signals and excitation at 594 nm and emission at 624 nm for interaction signals. The quantification of PLA signals was done by using the MetaMorph software 7.10.1.279 (Molecular Devices).

Immunoprecipitation

Cells were incubated in PBS with freshly prepared 0.5 mM DTSSP (3,3′-dithiobis (sulfosuccinimidyl propionate)) crosslinker (Cat. No. 21578, Thermo Fisher Scientific) or without DTSSP at room temperature for 15 min, followed by solubilization in lysis buffer containing 100 mM lactose for 30 min. Lysates (200 μg) were pre-cleared with protein G/A plus-agarose beads (Cat. No. sc-2002, Cat. No. sc-2001, Santa Cruz, TX, USA) for 2 hours, followed by immunoprecipitation with 3 μg mouse anti-TβRII (Cat. No. ab78419, Abcam) as previously described (ref. 5). Immune-complexes were eluted

with 2 × SDS protein sample buffer containing 0.125 mM Tris-HCl, pH 7.4, 4% SDS, and 20% glycerol. Lysates (20 μg) were used as input. Immunoprecipitates and input samples were examined by immunoblot analyses.

RAS activity assay

The activity of RAS small GTPases was determined using the Pan-RAS Activation Assay Kit (Cat. No. STA-400, Cell Biolabs, CA, USA), according to the manufacturer's instructions. Briefly, equivalent amounts of cell lysates and Raf1-RBD (Ras-binding domain) agarose beads were incubated to selectively pull down the active form of Ras. The precipitated GTP-Ras was eluted with 2 x SDS sample buffer and detected by immunoblot analysis using an anti-pan-Ras antibody (1: 1000, Cat. No. 240002, Cell Biolabs).

Intra-splenic injection and *in vivo* bioluminescence imaging

Experiments to assess the liver metastasis of tumors following intra-splenic injection of CRC cells were conducted as described previously (ref. 6). Briefly, 8-week-old NOD-SCID mice were anesthetized with 2,2,2-tribromoethanol (Avertin) (Cat. No. 75-80-9, Sigma-Aldrich, 250 mg/kg), and their left flanks were prepared for surgery. A small left abdominal flank incision was made, and the spleen was exteriorized for intra-splenic injection of galectin-8 Tet-off DLD1-Luc cells. A mixture of $5×10^6$ galectin-8 Tet-off DLD1-Luc cells/25 μL HBSS (Gibco) and Matrigel (Cat. No. 356231, BD Pharmingen, 25 μL) was prepared for one injection. Cells were then injected into the spleen using an Insulin syringe (Cat. No. 9151117S, B. Braun, Melsungen, Germany). To prevent leakage of cells and bleeding, a cotton swab was held over the site of injection for 1 min. The injected spleen was returned to the abdomen and the wound was sutured with 5-0 chromic catgut (Cat. No. CC125, UNIK, Taipei, Taiwan). The Kaplan-Meier

survival curve was calculated from mice treated with or without doxycycline (DOX, 200 μM). The Xenogen IVIS imaging system 200 (Caliper Life Sciences, MA, USA) was used to conduct a whole-body scan and evaluate the average radiance of the tumor mass (photons/second/cm^2/radian). The following conditions were used for image acquisition: open emission filter, exposure time = 60 s, binning = medium: 8, field of view = 26 x 26 cm, and f/stop = 1. Bioluminescence images were obtained weekly starting two weeks after implantation for the duration of the experiment to monitor liver metastasis. Mice were intraperitoneally injected with Xenolight D-luciferin potassium (150 mg/kg, Cat. No. 122799, PerkinElmer, MA, USA) 3 min before imaging, anesthetized (2% isoflurane) and placed in a light-tight camera box on the stage of the imaging chamber. Mice were imaged for 1 min with sequential 30-sec exposures. Bioluminescence images were quantified using the Living Image software 3.2 (PerkinElmer).

Luciferase reporter assay

We designed an *in vivo* monitoring system to investigate the formation of tumors by generating galectin-8 inducible DLD1 cells with luciferase expression. A month after luciferase lentivirus transduction, the luciferase activity was measured using a dual-luciferase reporter assay system (Cat. No. E1960, Promega), according to the manufacturer's instructions.

Histological analysis

Sections were stained with primary antibodies anti-galectin-8 (1:250, Cat. No. ab109519, Abcam), anti-B4GALT1 (1:200 dilution, Cat. No. ab121326, Abcam) and anti-B4GALT4 (1:200 dilution, Cat. No. ab169604, Abcam), followed by incubation with HRP-conjugated secondary antibodies. Digital light microscopic images were recorded

using Aperio XT (Leica Biosystems, Wetzlar, Germany) and manufactured by Aperio ImageScore 12.4.3. In the clinical patient samples, the results of B4GALT1 and B4GALT4 staining were classified according to the intensity and extent of staining. In the low expression group, either no staining was present (staining intensity score = 0) or positive staining was detected in less than 10% of the cells (staining intensity score = 1). In the high-expression group, positive immunostaining was present in 10%–30% (staining intensity score = 2) or more than 30% of cells (staining intensity score = 3). When staining clinical samples with galectin-8, the score was calculated as the product of the staining intensity and the percentage of positive cells. All IHC staining results were reviewed and independently scored by two pathologists. For experimental animal models, mice were sacrificed after processing and tissue samples were fixed in 10% formalin for 24 hours, embedded in paraffin, and cut into 3-μm-thick sections. Sections of liver and spleen were stained with the primary anti-galectin-8 antibody as well as hematoxylin and eosin as described previously (ref. 7).

Flow cytometry

To monitor rGal-8 binding with CRC cells, rGal-8 protein was labeled using a Lightning-link Fluorescein Labeling Kit (Cat. No. 707-0010, Innova Biosciences, Cambridge, UK), according to the manufacturer's instructions. The fluorescein-labeled rGal-8 was stored at 4°C to avoid light exposure. The binding of rGal-8 with CRC cells was determined by incubation of various doses of labeled rGal-8 with CRC cells on ice for 10 min. Annexin V staining was used to distinguish apoptotic CRC cells, according to a protocol described previously (ref. 8) using an APC-conjugated Annexin V staining kit (Cat. No. 550475, BD Pharmingen, CA, USA). Cell cycle analysis was conducted using DNA binding dye propidium iodide as described previously (ref. 9). PE-conjugated rat anti-TβRII primary antibody (Cat. No. 707-0010, BioLegend, CA, USA)

was used to determine the expression level of TβRII on the CRC cell surface. Cells were analyzed by a FACSCanto II Flow Cytometer (BD Biosciences). Cells were gated according to their light-scattering properties to exclude cell debris.

Wound healing

To investigate the wound-healing effect of recombinant galectin-8 on human CRC cells, the CRC cells were seeded in 6-well plates to grow as a confluent monolayer. The gaps were created by using the culture inserts (Cat. No. 81176, ibidi), according to the manufacturer's instructions. The cells were then treated with recombinant galectin-8, and dishes were photographed at the indicated time points using an Olympus IX71 microscope (Olympus Life Science). Wound areas were quantified by ImageJ software 1.8.0.

MTT (3-[4,5-dimethylthiazol-2-yl]-2,5-diphenyltratrazolium bromide) assay

The details of the MTT assay were described previously (ref. 10). Briefly, CRC cells were seeded at 1×10^5 cells/mL in 96-well plates and incubated overnight before treatment, followed by incubation for 48 hours, or the indicated time points. Cells were then treated with MTT (Cat. No. M5655, Sigma-Aldrich), according to the manufacturer's instructions. Four hours later, the absorbance of each well was measured at 450 nm using a Spectra Max M2 microplate reader (Molecular Devices, CA, USA).

Caspase activity

Caspase activity was measured using the Sensolyte AFC Caspase Substrate Sampler Kit (Cat. No. 71117, AnaSpec, CA, USA), and cell lysates from different time points after treatment were collected according to the manufacturer's instructions.

Fluorescence was measured using a Spectra Max M2 microplate reader using excitation and emission wavelengths of 380 and 500 nm, respectively. All data were calculated in units of micromolar AFC per hour based on a standard AFC curve (ranging between 0.057–57 μM). Etoposide (10 μM), an inducer of apoptosis (ref. 11), was used as a positive control.

Statistical analysis

All results are presented as means ± SD. The comparison of data between two groups was performed using an unpaired Student's *t*-test, while the results concerning more than two parameters were subjected to a one-way ANOVA. A log-rank test was used to compare the distribution of the time to occurrence of an event of interest in independent groups. All tests were two-tailed, and a p-value of less than 0.05 was considered statistically significant (*p ≤ 0.05, **p ≤ 0.01, ***p ≤ 0.001, ****P ≤ 0.0001). The figure panel and legends indicate the number of animals in the survival curve, recorded ROI or independent experiments. The sample size was determined based on previous studies (ref. 6) and animals were randomly assigned to experimental groups. During the immunohistochemical staining experiments, the investigators remained blind to both the clinical samples and the mouse treatment group. All statistical analyses were performed using GraphPad Prism 10 software.

Supplementary References

1. Li CW, Hon KW, Ghosh B, Li PH, Lin HY, Chan PH, et al. Synthesis of oligomeric mannosides and their structure-binding relationship with concanavalin A. Chem Asian J. 2014;9(7):1786-96.

2. Tsai CM, Guan CH, Hsieh HW, Hsu TL, Tu Z, Wu KJ, et al. Galectin-1 and galectin-8 have redundant roles in promoting plasma cell formation. J Immunol. 2011;187(4):1643-52.

3. Moore CB, Guthrie EH, Huang MT, Taxman DJ. Short hairpin RNA (shRNA): design, delivery, and assessment of gene knockdown. Methods Mol Biol. 2010;629:141-58.

4. Han H. RNA Interference to Knock Down Gene Expression. Methods Mol Biol. 2018;1706:293-302.

5. Bonifacino JS, Dell'Angelica EC, Springer TA. Immunoprecipitation. Curr Protoc Immunol. 2001;Chapter 8:Unit 8.3.

6. Lee WY, Hong HK, Ham SK, Kim CI, Cho YB. Comparison of colorectal cancer in differentially established liver metastasis models. Anticancer Res. 2014;34(7):3321-8.

7. Cardiff RD, Miller CH, Munn RJ. Manual hematoxylin and eosin staining of mouse tissue sections. Cold Spring Harb Protoc. 2014;2014(6):655-8.

8. Zhang G, Gurtu V, Kain SR, Yan G. Early detection of apoptosis using a fluorescent conjugate of annexin V. Biotechniques. 1997;23(3):525-31.

9. Krishan A. Rapid flow cytofluorometric analysis of mammalian cell cycle by propidium iodide staining. J Cell Biol. 1975;66(1):188-93.

10. Carmichael J, DeGraff WG, Gazdar AF, Minna JD, Mitchell JB. Evaluation of a tetrazolium-based semiautomated colorimetric assay: assessment of

chemosensitivity testing. Cancer Res. 1987;47(4):936-42.

11. Karpinich NO, Tafani M, Rothman RJ, Russo MA, Farber JL. The course of etoposide-induced apoptosis from damage to DNA and p53 activation to mitochondrial release of cytochrome c. J Biol Chem. 2002;277(19):16547-52.

Supplementary Table

Supplementary Table 1. The sequences of primers for qPCR

Primer name	Sequence
FUT8 qPCR _F:	5'- GACAGAACTGGTTCAGCGGAGA -3'
FUT8 qPCR _R:	5'- GCAGTAGACCACATGATGGAGC -3'
MGAT1 qPCR _F:	5'- CCTATGACCGAGATTTCCTCGC -3'
MGAT1 qPCR _R:	5'- TGAAGCTGTCCCTGCCCGTATA -3'
MGAT2 qPCR _F:	5'- CCAGGGAATTGACAACGTCCTC -3'
MGAT2 qPCR _R:	5'- AGGGTCACTACCTGGAAACTCG -3'
MGAT3 qPCR _F:	5'- ACGTCAACCACGAGTTCGACCT -3'
MGAT3 qPCR _R:	5'- AAGCCGTGAAGTTGGACTCGCA -3'
MGAT4a qPCR _F:	5'- TCTACCAAGGGCATACGCTGGA -3'
MGAT4a qPCR _R:	5'- GATGTTCTTGGTTGCCGCTATGG -3'
MGAT5 qPCR _F:	5'- AGGCAGAACCAGTCCCTTGTGT -3'
MGAT5 qPCR _R:	5'- CTTTGTGCTGGAGCCATAAACAG -3'
B4GALT1 qPCR _F:	5'- GTATTTTGGAGGTGTCTCTGCTC -3'
B4GALT1 qPCR _R:	5'- GGGCGAGATATAGACATGCCTC -3'
B4GALT2 qPCR _F:	5'- GACCGCGACAAGCATAACGAAC -3'

B4GALT2 qPCR _R:	5’- AGACACCTCCAAGACCTGGTAC -3’
B4GALT3 qPCR _F:	5’- TCCTCAAGGTCTGCCCTACTGT -3’
B4GALT3 qPCR _R:	5’- ATTCCGCTCCACAATCTCTGCC -3’
B4GALT4 qPCR _F:	5’- CTCTGACTAATGAAGCATCCACG -3’
B4GALT4 qPCR _R:	5’- CTGCCTGTACCTCTTCCAAAGTG -3’
B4GALT5 qPCR _F:	5’- GAAGATGACGACCTCTGGAACAG -3’
B4GALT5 qPCR _R:	5’- GCCGTTCTTTTGACTTCCTCAGC -3’
B4GALT6 qPCR _F:	5’- CTCATTCCTTTCCGTAATCGCCA -3’
B4GALT6 qPCR _R:	5’- GCCCACATTGAAAAGCATCGCAC -3’
B4GALT7 qPCR _F:	5’- TGCTCAACCAGGTGGACCACTT -3’
B4GALT7 qPCR _R:	5’- AGGTCAACGTCGTGCATGGCAA -3’
LGALS1 qPCR _F:	5’- TTGTGGTCTGGTCGCCAGC -3’
LGALS1 qPCR _R:	5’- TCAGTCAAAGGCCACACATTT -3’
LGALS2 qPCR _F:	5’- GGGTCAGAGGTCAAGTTCACAGT -3’
LGALS2 qPCR _R:	5’- CCATCTGGCAGCTTCACCTT -3’
LGALS3 qPCR _F:	5’- GTGCCTTATAACCTGCCTTTGC -3’
LGALS3 qPCR _R:	5’- TCACCGTGCCCAGAATTGTT -3’
LGALS4 qPCR _F:	5’- GCCTGCCCACCATGGA -3’
LGALS4 qPCR _R:	5’- TTGCAGCCTCCCGAAATATG -3’

LGALS8 qPCR _F:	5'- CCCGGTAATCCCGTTTGTT -3'
LGALS8 qPCR _R:	5'- TGCCCACGTATCACAATCAAAG -3'
LGALS9 qPCR _F:	5'- CCATTACCCAGACAGTCATCCA -3'
LGALS9 qPCR _R:	5'- GATGGCGGGAGTAGAGAACATC -3'
MMP2 qPCR _F:	5'- AGCGAGTGGATGCCGCCTTTAA -3'
MMP2 qPCR _R:	5'- CATTCCAGGCATCTGCGATGAG -3'
MMP7 qPCR _F:	5'- TCGGAGGAGATGCTCACTTCGA -3'
MMP7 qPCR _R:	5'- GGATCAGAGGAATGTCCCATACC -3'
MMP9 qPCR _F:	5'- GCCACTACTGTGCCTTTGAGTC -3'
MMP9 qPCR _R:	5'- CCCTCAGAGAATCGCCAGTACT -3'

Supplementary Legend

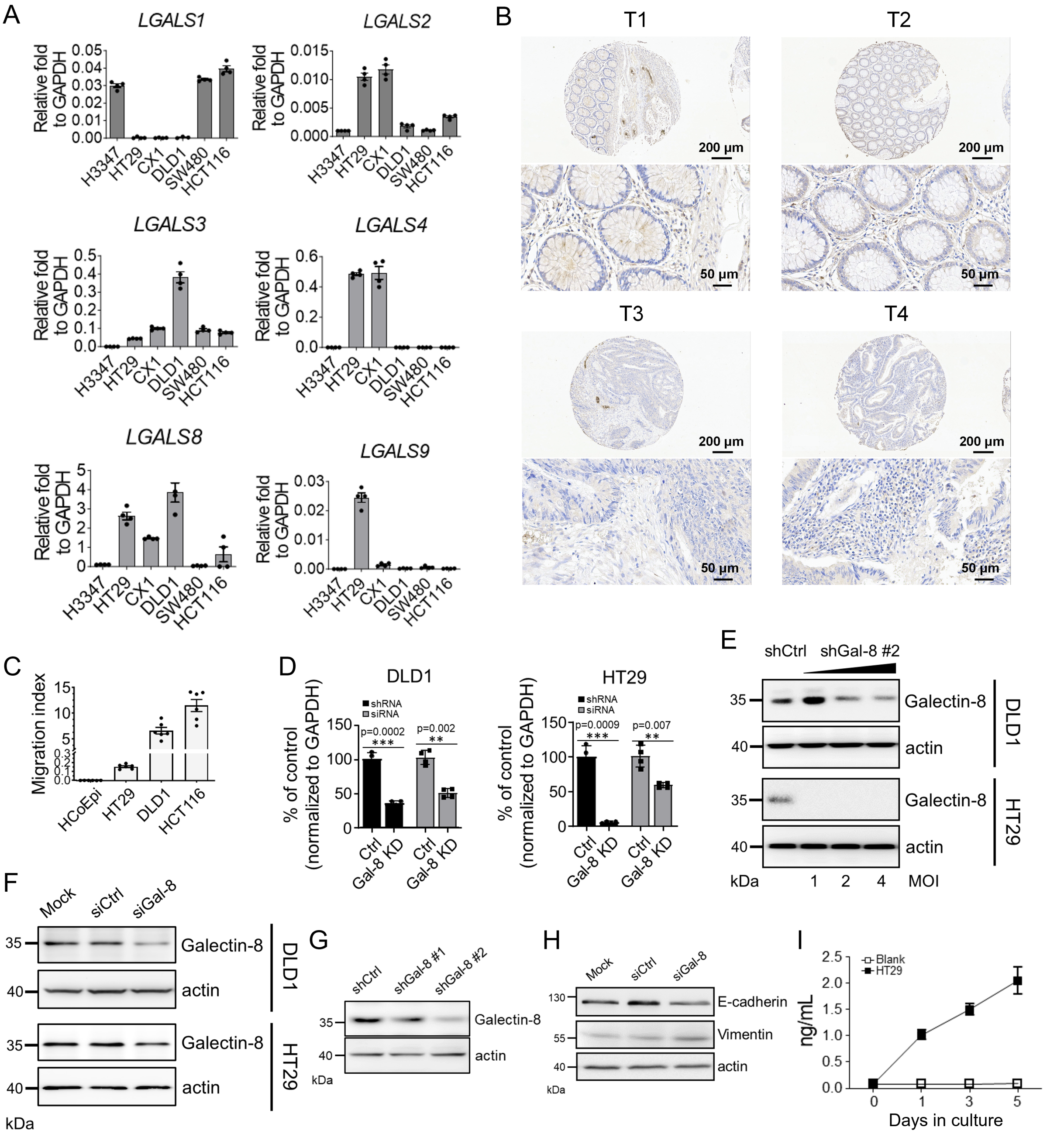

Supplementary Figure 1. Expression of *LGALS8* in CRC cells and determination of knockdown efficiency of shRNAs and siRNAs targeting *LGALS8*. (A) RT-qPCR shows the mRNA levels of the indicated galectins in six CRC cell lines. Results are shown as mean ± SD (n = 4). Two biological replicates were performed. (B) IHC staining of galectin-8 expression in tumor tissue from CRC patients, classified according to the T-classification. (C) Cell migration activities of HCoEpi, DLD1, HT29,

and HCT116 cells analyzed by xCELLigence RTCA at 48 hours. (D) RT-qPCR shows the mRNA level of galectin-8 in CRC cells expressing shGal-8 #2 or treated with a mixture of three distinct siRNA sequences against Gal-8. (E) Knockdown efficiency of galectin-8 by different amounts of shGal-8 (shGal-8#2) in DLD1 and HT29 cells at 120 hours post-transduction. (F) Knockdown efficiency of galectin-8 by siRNAs in DLD1 and HT29 cells after 48 hours of treatment. (G) The efficiency of knockdown of *LGALS8* by two independent shRNAs by lentiviral transduction in HT29 cells. (H) Western blotting shows the expression of EMT markers in mock-, siCtrl-, and siGal-8-treated HT29 cells. (I) ELISA showing TGF-β levels in the culture medium of HT29 cells at the indicated days after plating. Results in A, B, C, and H are shown as mean ± SD (2 biological replicates with technical duplicates for A, C, and H, and 3 biological replicates with technical duplicates for B). Statistical tests were calculated by *t*-test. ns, not significant.

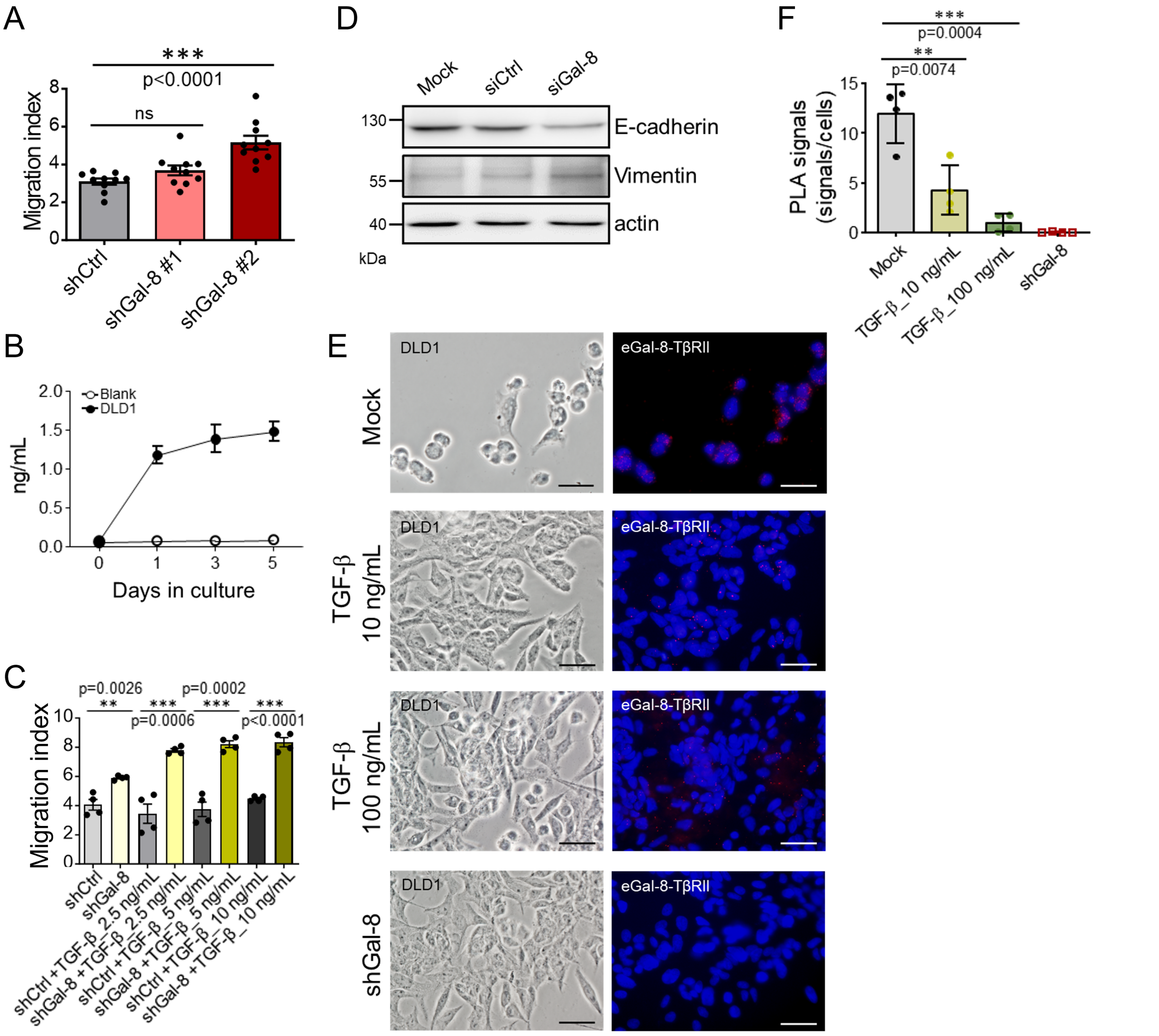

Supplementary Figure 2. Knockdown of *LGALS8* promotes TGF-β response in DLD1 cells. (A) Cell migration of DLD1 cells transduced with shCtrl or two different shRNAs against galectin-8 analyzed by xCELLigence RTCA 48 hours after transduction. (B) Cell migration activities of shCtrl- and shGal-8-expressing cells treated with TGF-β at the indicated doses analyzed by xCELLigence RTCA 48 hours after treatment. (C) Western blotting shows the expression of EMT markers in mock-, siCtrl-, and siGal-8-treated DLD1 cells. (D) ELISA showing TGF-β level in the culture medium of DLD1 cells at the indicated days after plating. (E, F) PLA images (E) and statistical analysis of PLA signal quantification (F) show the effects of TGF-β on the interaction of endogenous galectin-8 (eGal-8) with TβRII. DAPI staining was used to

label cell nuclei. Scale bar = 20 μm. Results in A, B, C, and F are shown as mean ± SD (2 biological replicates with technical quintuplicates for A; 2 biological replicates with technical duplicates for each in B and C; 4 biological replicates for F). Statistical tests are calculated by *t*-test (A, B, D, F, and H). ns, not significant.

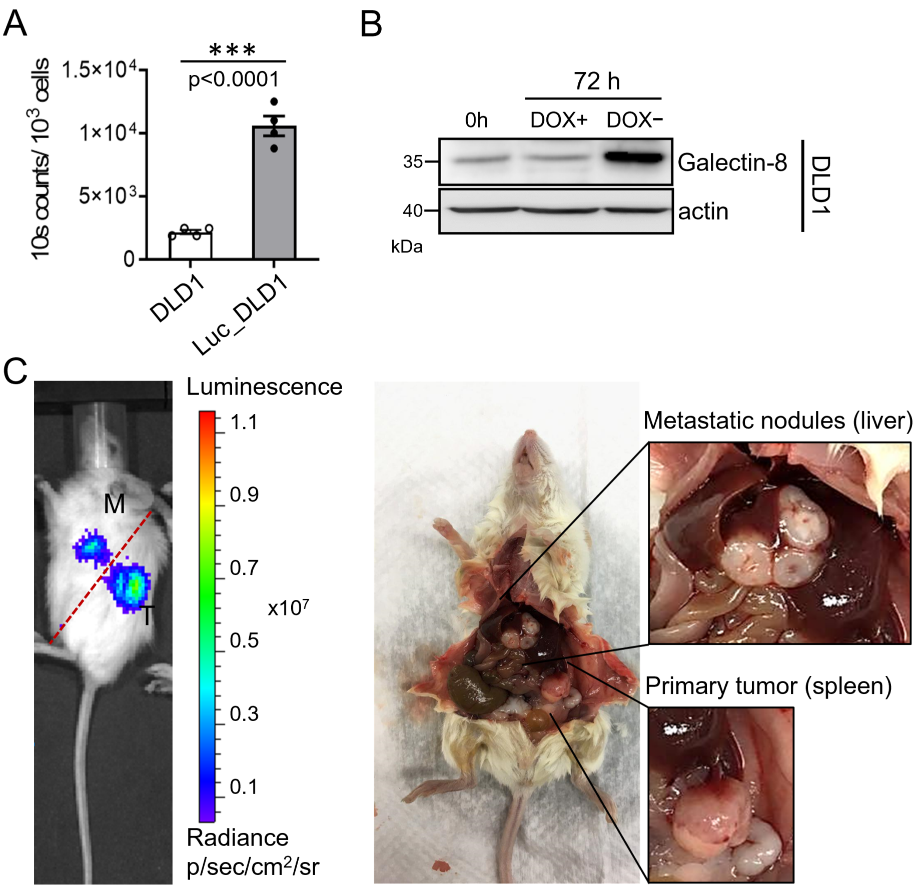

Supplementary Figure 3. Induction of intrasplenic tumors and liver metastases by implantation of luciferase-expressing galectin-8 Tet-off DLD1 cells in NOD-SCID mice. (A) Luciferase activities from DLD1 cells stably expressing luciferase. Results are shown as mean ± SD (2 biological replicates in duplicates). Statistical tests were calculated by *t*-test. (B) Western blot shows the induction of galectin-8 in DLD1 cells expressing luciferase 72 hours after removal of DOX (DOX−) compared with DOX-treated cells (DOX+, 200 μM). (C) Left, bioluminescence images of a xenografted NOD-SCID mouse (left) after 21 days of treatment with DOX (200 μM). Right, gross anatomy showing the presence of primary and metastatic tumors in the spleen and liver, respectively.

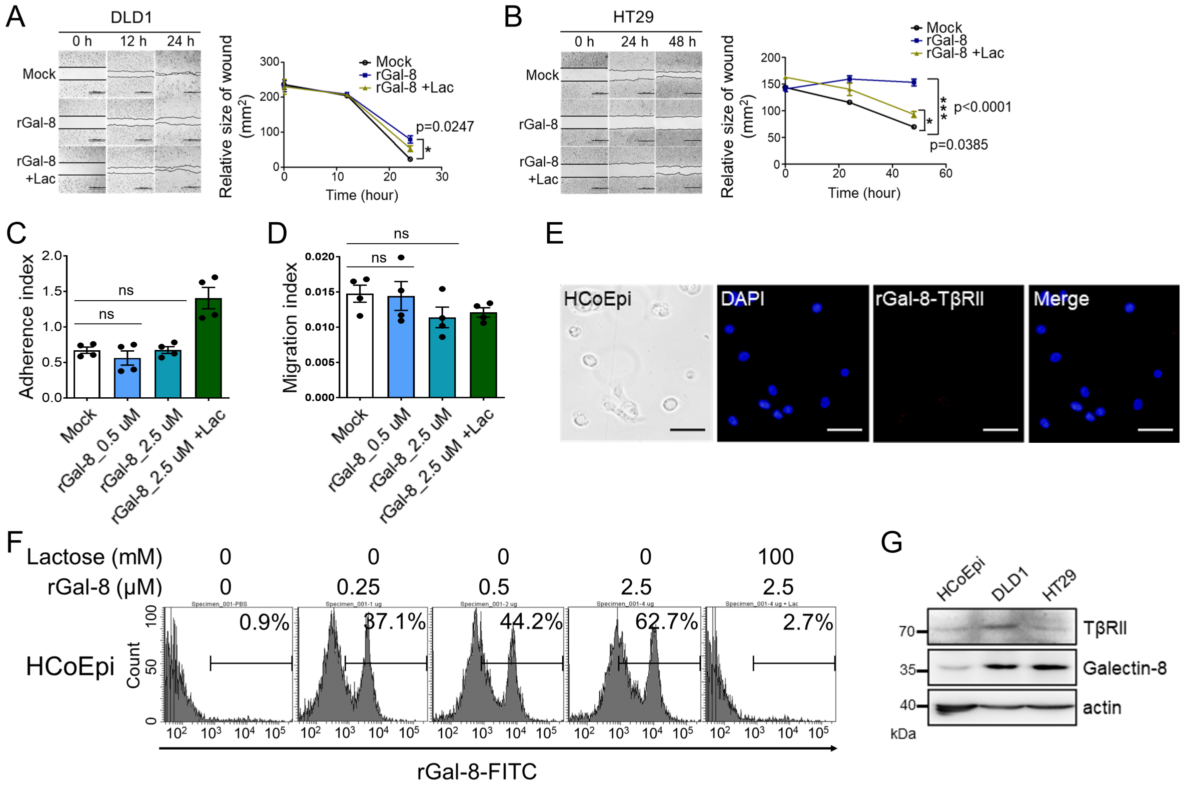

Supplementary Figure 4. The effect of rGal-8 on CRC cells and normal human intestinal epithelial cells. (A) DLD1 and (B) HT29 cells treated with rGal-8 (2.5 μM) or rGal-8 + lactose (Lac, 100 mM) or left untreated (mock) were subjected to wound-healing assays. The images on the left show the wounds at 0 hour and the wound status at the indicated time points after the scratching. The wound-healing area was measured by calculating the wound area at each time point (right). Scale bar = 500 μm. (C, D) Cell adhesion (C) and migration (D) activities of normal human intestinal epithelial (HCoEpi) cells treated with rGal-8 at the indicated doses in the absence or presence of lactose (Lac, 100 mM), analyzed by xCELLigence RTCA 6 hours (C) and 48 hours (D) after treatment. (E) PLA shows the absence of interaction between rGal-8 and TβRII in HCoEpi cells. Scale bar = 20 μm. (F) FACS shows the binding level of FITC-conjugated rGal-8 with HCoEpi cells at the different doses; the binding was blocked by lactose treatment. (G) Western blot analysis of TβRII expression in HCoEpi, DLD1, and HT29 cells. Results in A–D are shown as mean ± SD (2

biological replicates with technical duplicates). Statistical tests were calculated by ANOVA for A and B, *t*-test for C and D. ns, not significant.

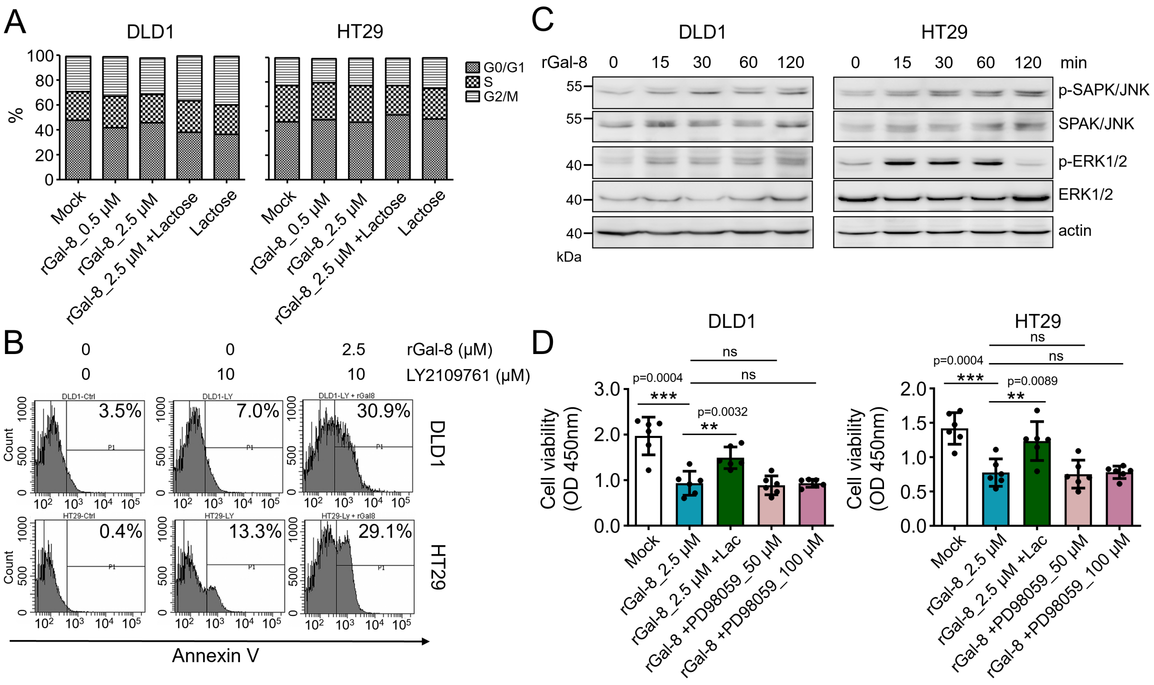

Supplementary Figure 5. The effect of rGal-8 on the cell cycle and apoptosis of CRC cells. (A) Cell cycle distribution of DLD1 and HT29 cells after treatment with rGal-8 at the indicated doses for 72 hours. Lactose (100 mM) was added in some groups. (B) FACS shows the percentage of apoptotic (Annexin V$^+$) cells among DLD1 and HT29 cells treated with LY2109761 (10 μM) alone or together with rGal-8 (2.5 μM) for 24 hours. (C) Western blot analysis of the expression of phosphorylated and total JNK and ERK1/2 in DLD1 and HT29 cells treated with rGal-8 (2.5 μM) at the indicated time points after 30 minutes of treatment with LY2109761 (10 μM). (D) Cell viability of DLD1 and HT29 cells treated for 72 hours with rGal-8 (2.5 μM) alone or with the ERK inhibitor PD98059 at the indicated doses. Results in D are shown as mean ± SD (three independent experiments performed in duplicates). Statistical tests were calculated by *t*-test.

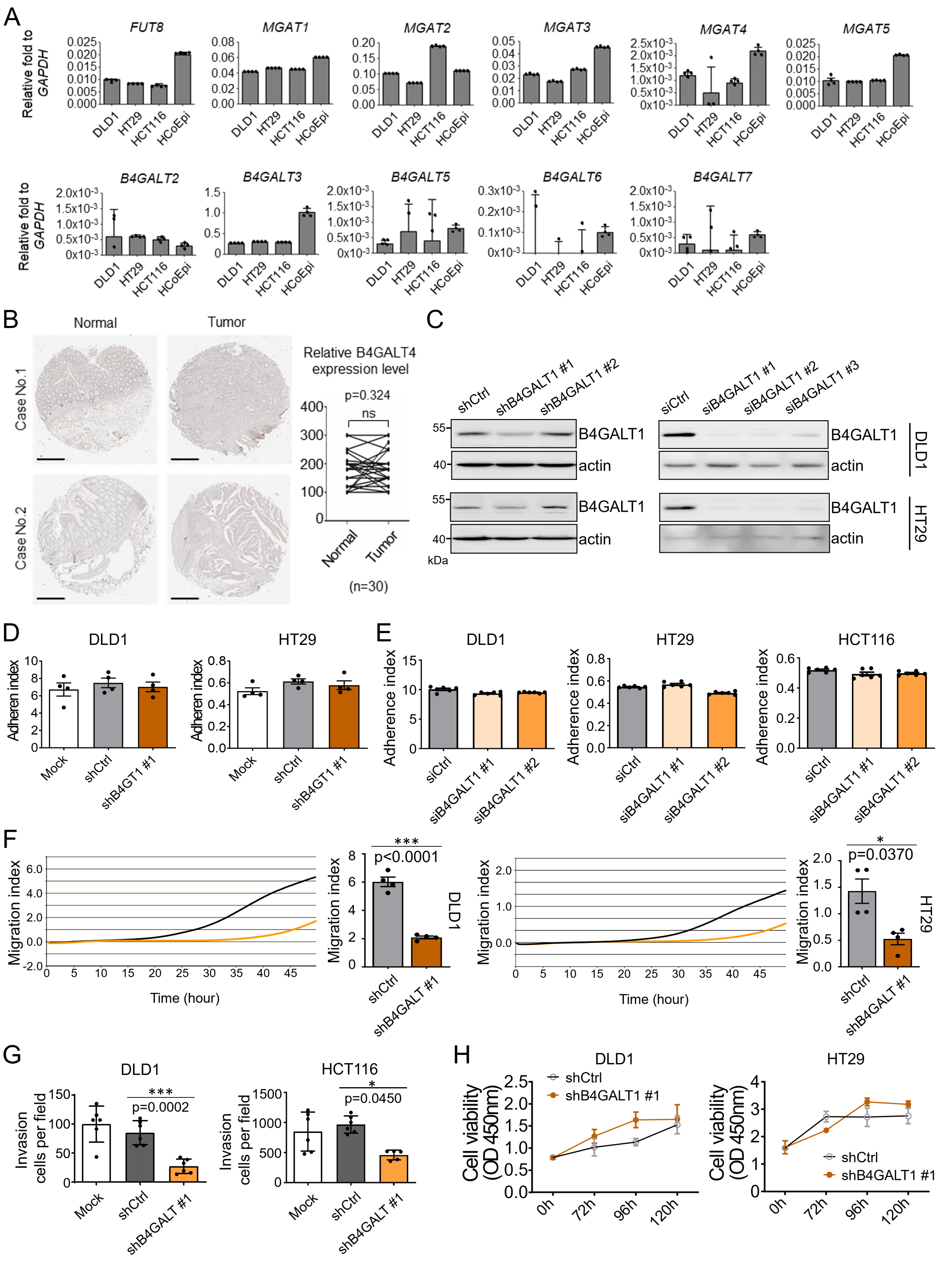

Supplementary Figure 6. Knockdown of *B4GALT1* reduces invasive and migratory activities of CRC cells but does not affect cell adhesion and viability. (A) RT-qPCR shows the mRNA levels of several genes involved in N-glycosylation in

three CRC cells and the normal human intestinal epithelial HCoEpi cells. The genes studied are as follows: α-1,6- fucosyltransferase (*FUT8*), mannosyl (α-1,3-)-glycoprotein β-1,2-N-acetylglucosaminyltransferase (*MGAT1*), mannosyl (α-1,6-)-glycoprotein β-1,2-N-acetylglucosaminyltransferase (*MGAT2*), mannosyl (β-1,4-)-glycoprotein β-1,4-N-acetylglucosaminyltransferase (*MGAT3*), mannosyl (α-1,3-)-glycoprotein β-1,4-N-acetylglucosaminyltransferase (*MGAT4*), mannosyl (α-1,6-)-glycoprotein β-1,6-N-acetylglucosaminyltransferase (*MGAT5*), and β-1,4-galactosyltransferases 2–7 (*B4GALT2-7*). (B) IHC staining of B4GALT4 levels in paired non-tumor and tumor tissues from CRC patients (left). The right panel shows the distribution of immunoactivity scores of B4GALT4 expression in normal colon and primary CRC tissues (n=30). ns, not significant. Scale bar = 300 µm. (C) Knockdown efficiency of B4GALT1 by lentiviral transduction (left) and siRNA transfection (right) in DLD1 and HT29 cells. Two independent shRNAs and three independent siRNAs were used against B4GALT1. (D, E) The effect of knockdown of *B4GALT1* by shRNA (B) or siRNA (C) on adhesion of the indicated CRC cells 6 hours after plating. Results were analyzed by xCELLigence RTCA. (F) The effect of knockdown of *B4GALT1* by shRNA on the migratory activity of the indicated CRC cells. The results were analyzed by xCELLigence RTCA at 48 hours. Results in A and D−H are shown as mean ± SD (2 biological replicates with technical duplicates for each in A, D, F, and H, and 3 biological replicates with technical duplicates for E and G). Statistical tests were calculated by *t*-test. ns, not significant.

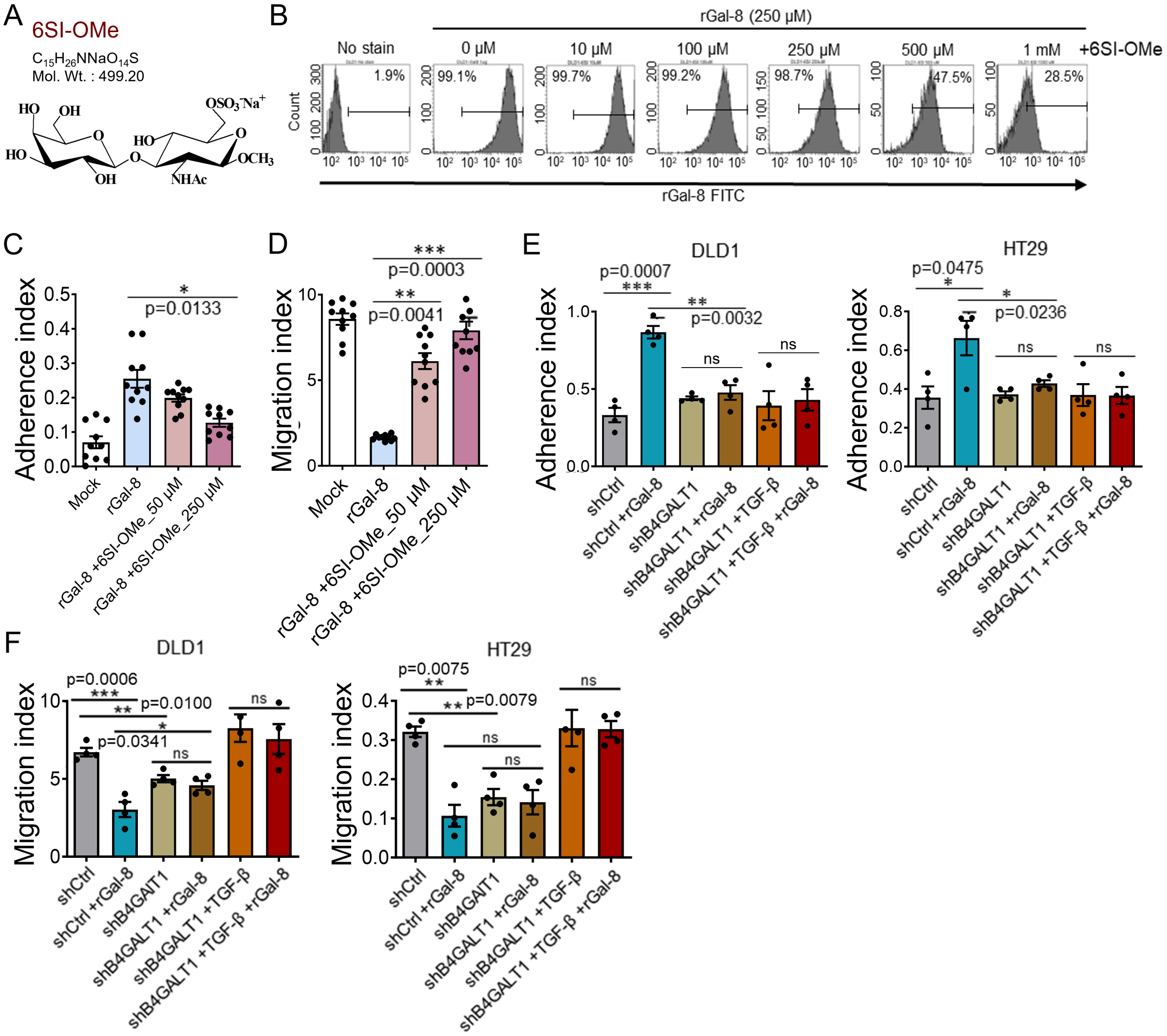

Supplementary Figure 7. Knockdown of *B4GALT1* abrogates the anti-metastatic effects mediated by rGal-8. (A) Structure and molecular weight of the galectin-8 inhibitor, 6SI-OMe. (B) FACS shows the dose-response of 6SI-Ome (250 μM) to block the binding of FITC-conjugated rGal-8 (2.5 μM) with DLD1 cells. (C, D) Cell adhesion (C) and migration (D) activities of DLD1 cells treated with rGal-8 (2.5 μM) alone or together with 6SI-OMe (50 μM or 250 μM) analyzed by xCELLigence RTCA 6 hours (C) and 48 hours (D) after treatment. (E, F) Cell adhesion (E) and migration (F) activities of DLD1 or HT29 cells expressing shCtrl or shB4GALT1 in the presence or absence of rGal-8 (2.5 μM) and/or TGF-β (100 ng/mL) analyzed by xCELLigence RTCA 6 hours (E) and 48 hours (F) after treatment. Results in C–F are shown as

mean ± SD (2 biological replicates with technical quintuplicates for C and D, and 2 biological replicates with technical duplicates for E and F). Statistical tests were calculated by *t*-test. ns, not significant.

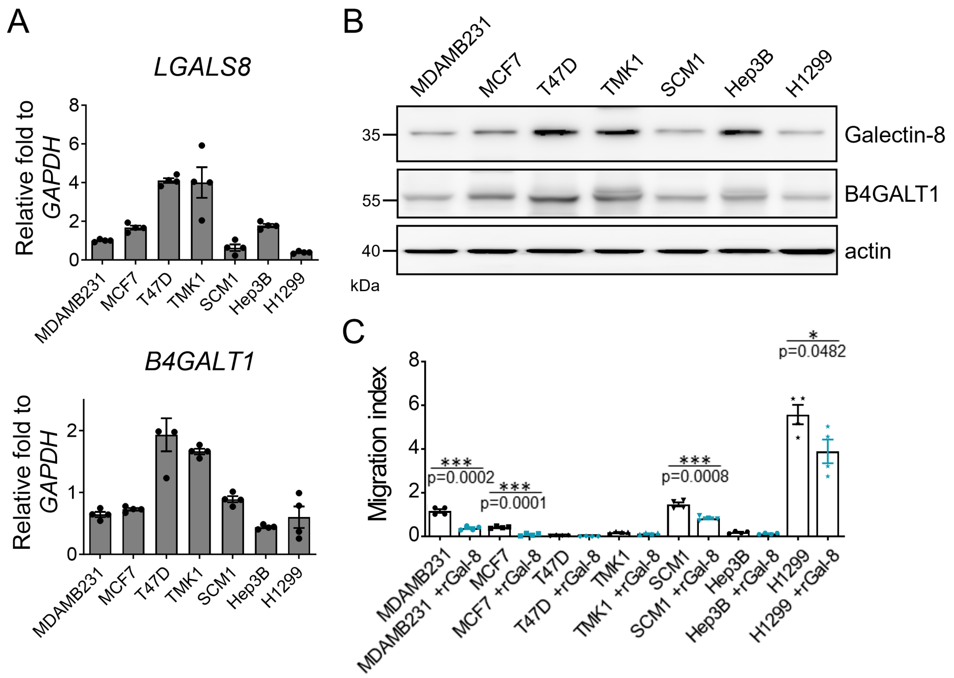

Supplementary Figure 8. The anti-metastatic effects of rGal-8 in other types of cancer cells. (A) RT-qPCR shows mRNA levels of galectin-8 and B4GALT1 in breast (MDAMB231, MCF7, and T47D), gastric (TMK1 and SCM1), liver (Hep3B), and lung (H1299) cancer cell lines. (B) Western blotting shows protein expression of galectin-8 and B4GALT1 in different cancer cell lines. Actin was used as a protein loading control. (C) Cell migration activities of different cancer cells in the absence or presence of rGal-8 (2.5 μM) analyzed by the xCELLigence RTCA 48 hours after treatment. Results in A and C are shown as mean ± SD (2 biological replicates with technical duplicates). Statistical tests were calculated by *t*-test. ns, not significant.

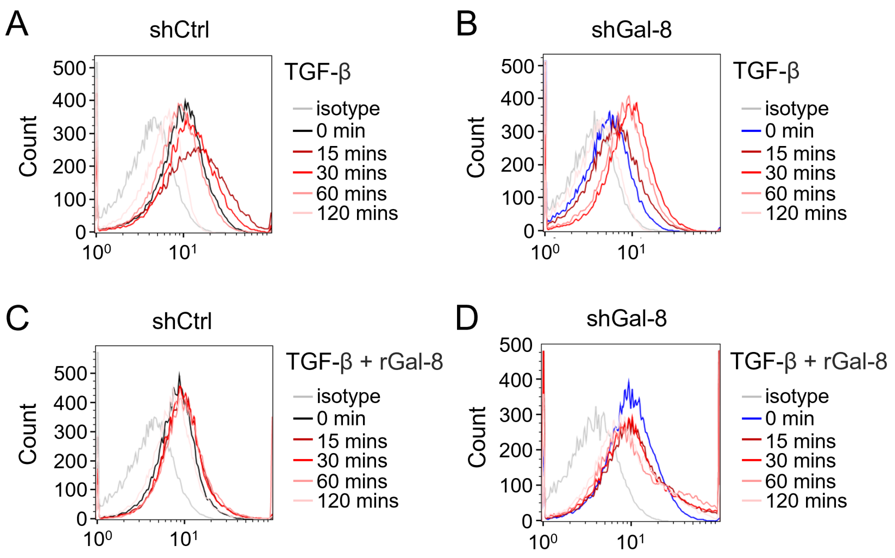

Supplementary Figure 9. Cell surface expression of TβRII in the presence or absence of galectin-8 and TGF-β. FACS shows the cell surface levels of TβRII on DLD1 cells expressing shCtrl (A, C) or shGal-8 (B, D) treated with TGF-β (100 ng/mL) (A, B) or TGF-β + rGal-8 (2.5 μM) (C, D) at the indicated time points. The gray histogram indicates the background binding of the isotype control antibody.
