# Supplementary material for: B4GALT1-dependent galectin-8 binding with TGF-β receptor suppresses colorectal cancer progression and metastasis
Source: Cell Death Dis. 2024 Sep 4;15(9):654. doi: 10.1038/s41419-024-07028-3 (PMC11375092; doi:10.1038/s41419-024-07028-3)

Raw images of immunoblotting experiments

Figure 1A

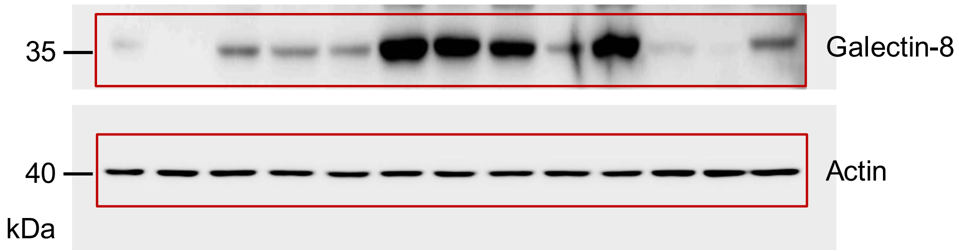

Figure 1D

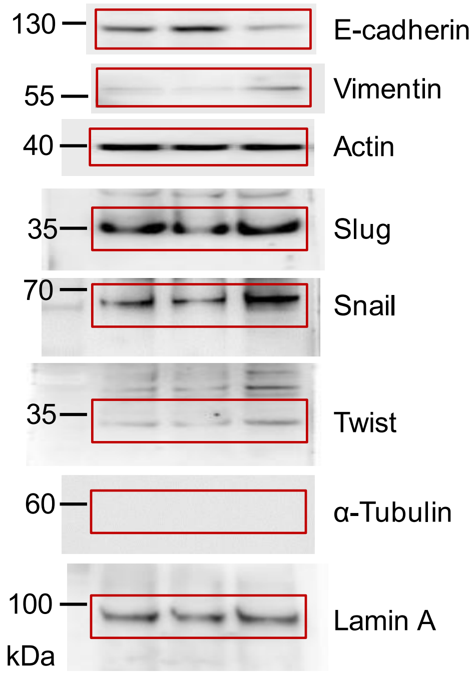

Figure 1H

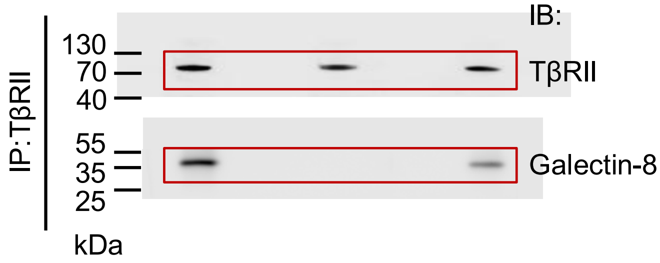

Figure 1I

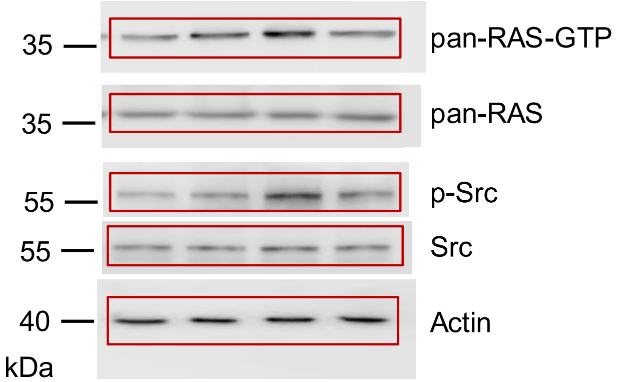

Figure 2D

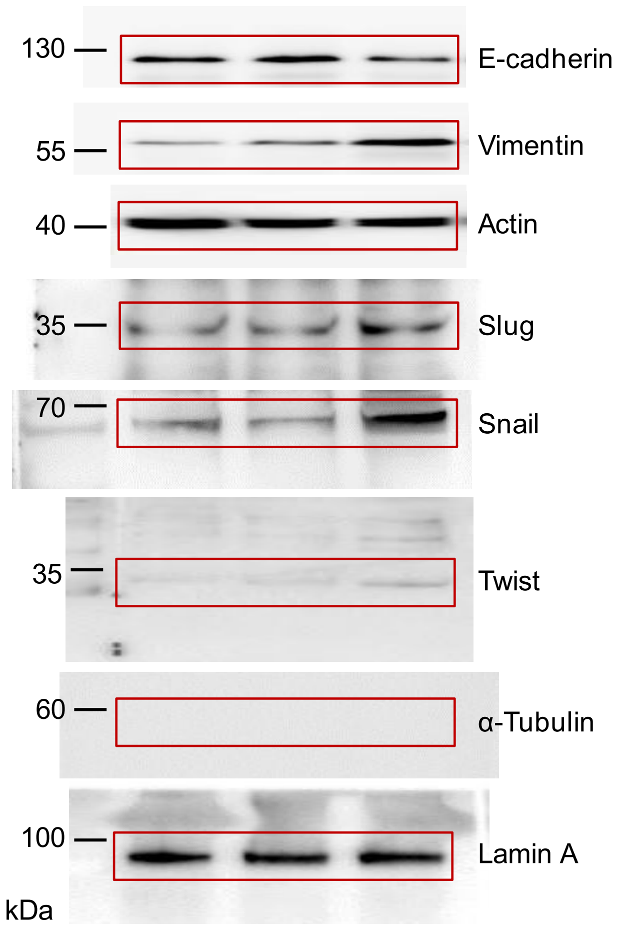

Figure 2H

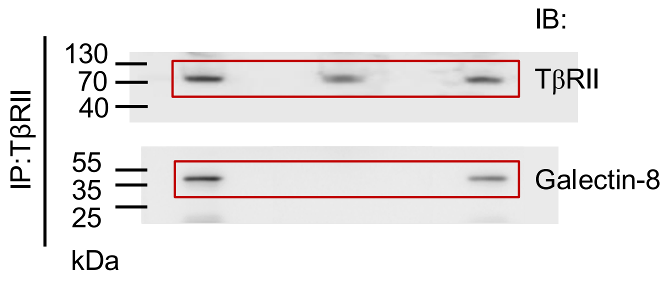

Figure 2K

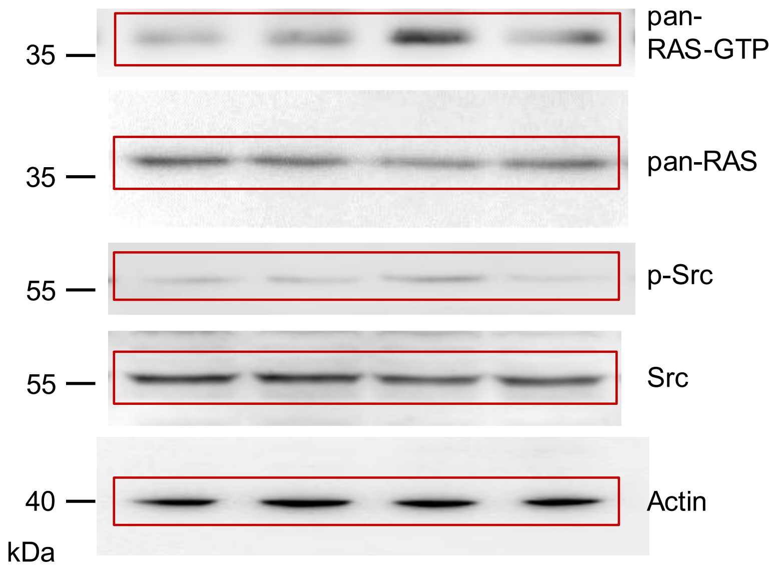

Figure 3F

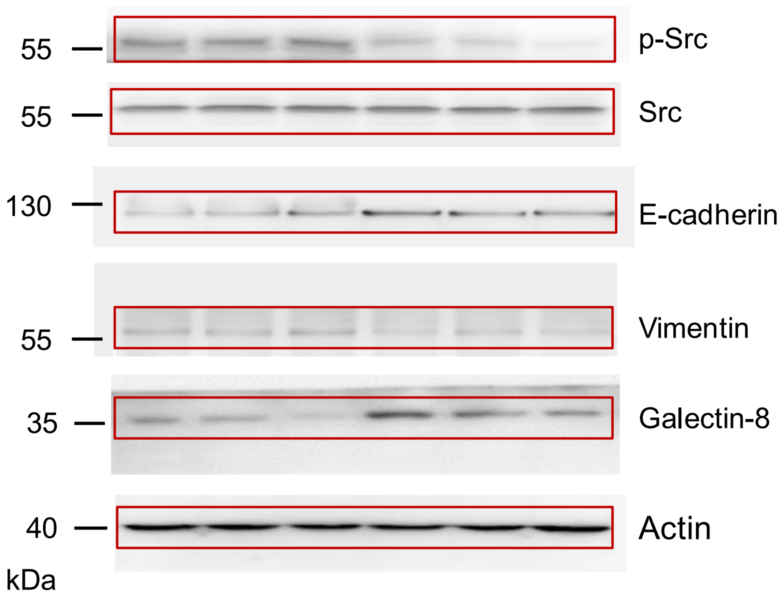
55
p-Src
55
Src
130
E-cadherin
55
Vimentin
35
Galectin-8
40
Actin
kDa

Figure 4C

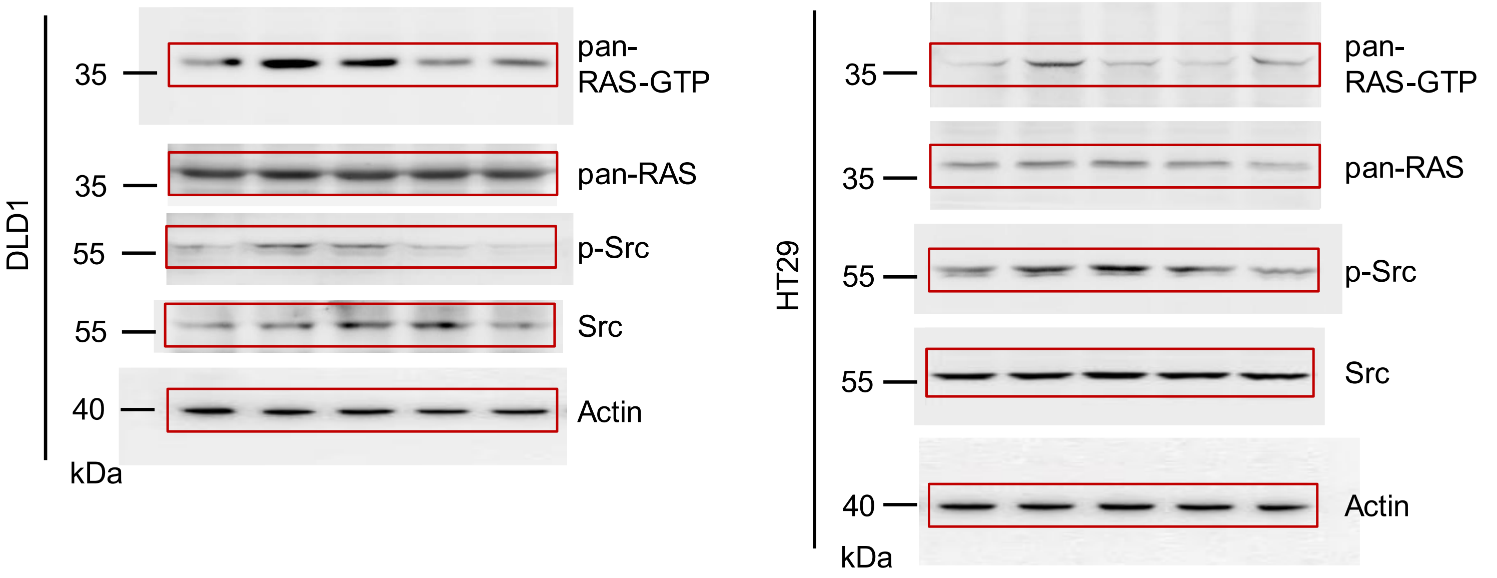

DLD1
35
pan-
RAS-GTP
35
pan-RAS
55
p-Src
55
Src
40
Actin
kDa
HT29
35
pan-
RAS-GTP
35
pan-RAS
55
p-Src
55
Src
40
Actin
kDa

Figure 5F

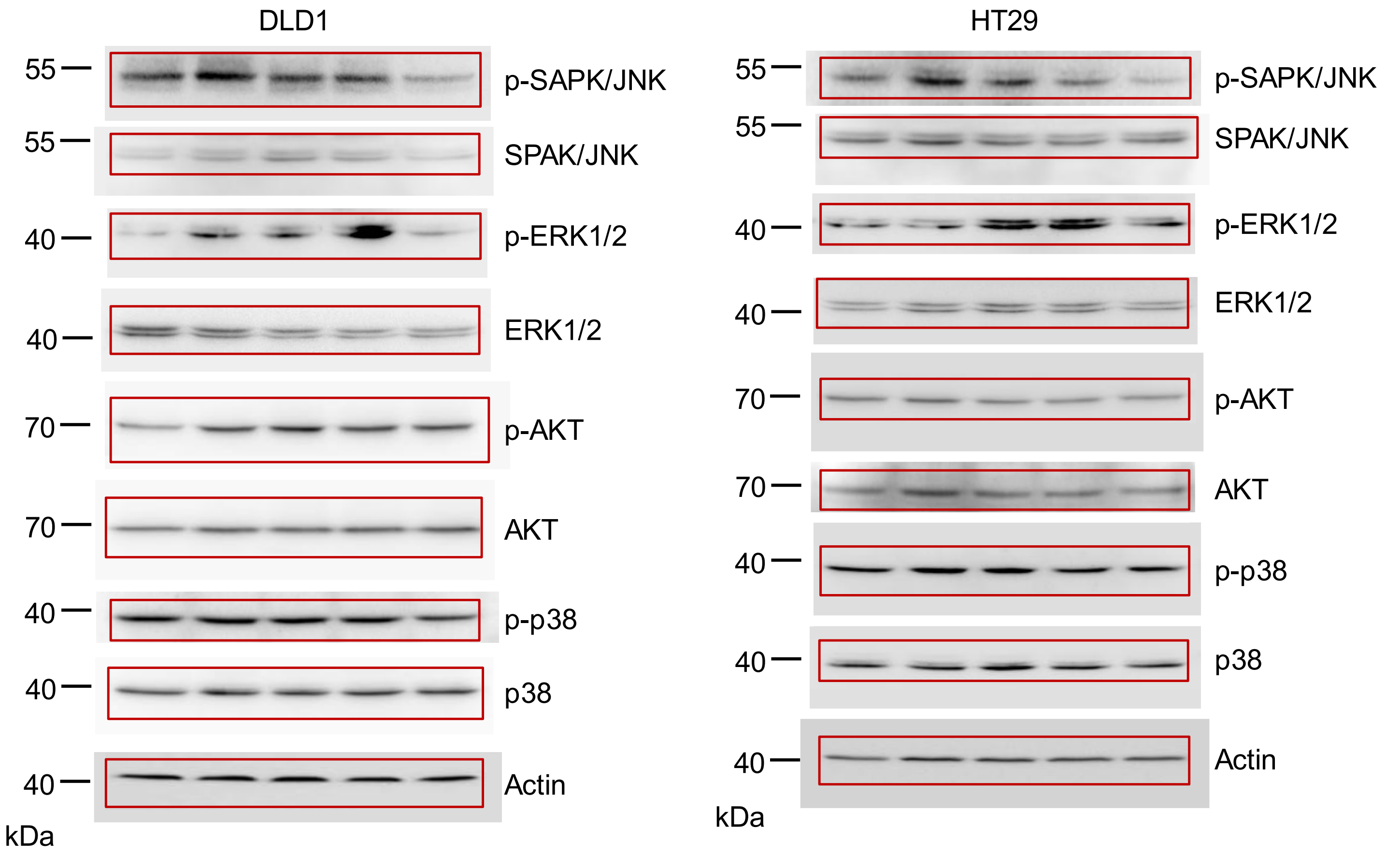

DLD1
HT29
55
p-SAPK/JNK
55
SPAK/JNK
40
p-ERK1/2
40
ERK1/2
70
p-AKT
70
AKT
40
p-p38
40
p38
40
Actin
kDa

Figure 6B

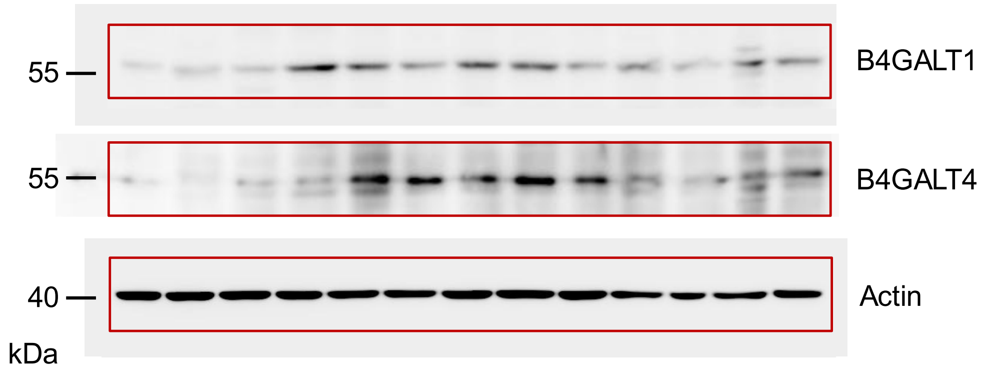

Figure 6J

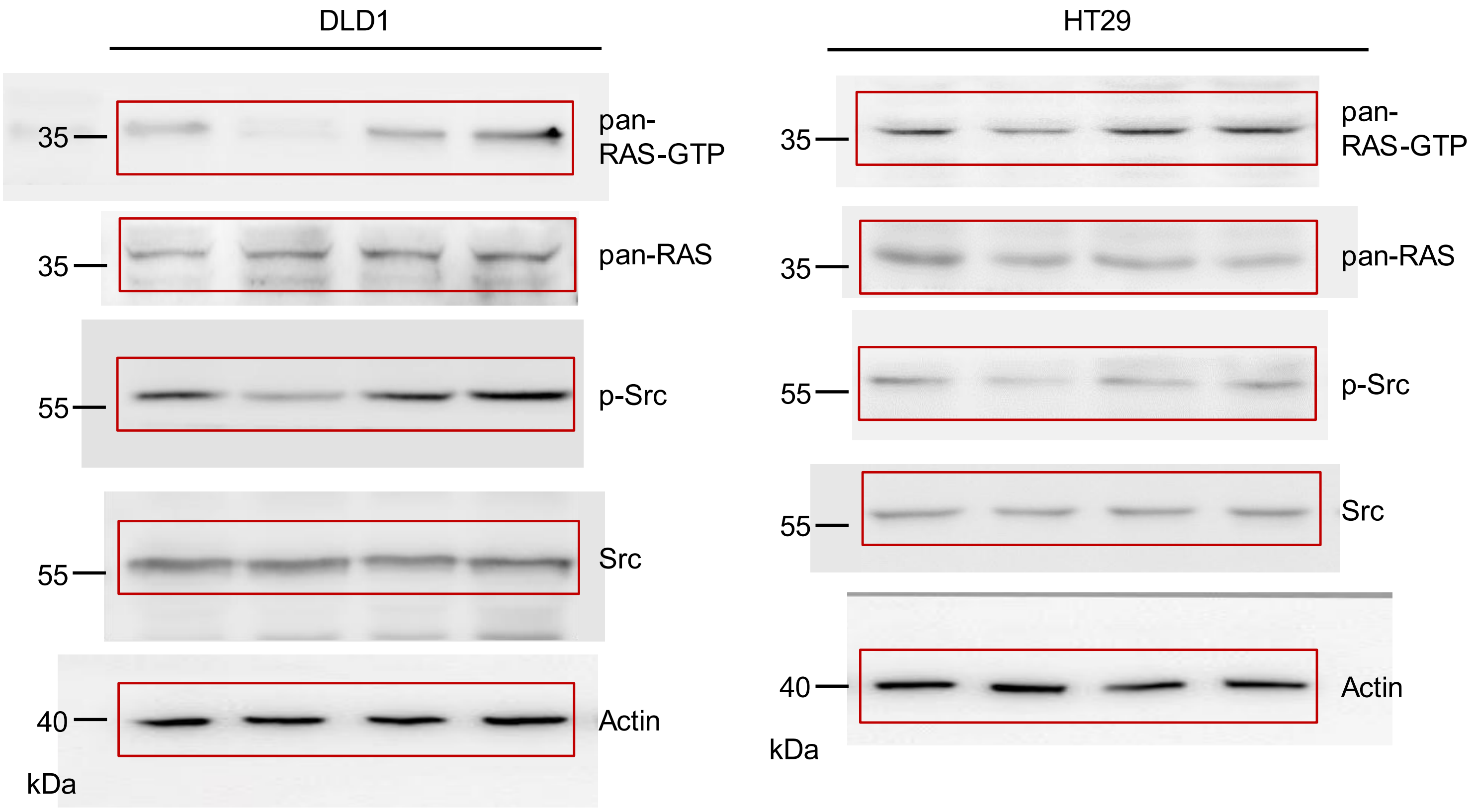

Figure S1D

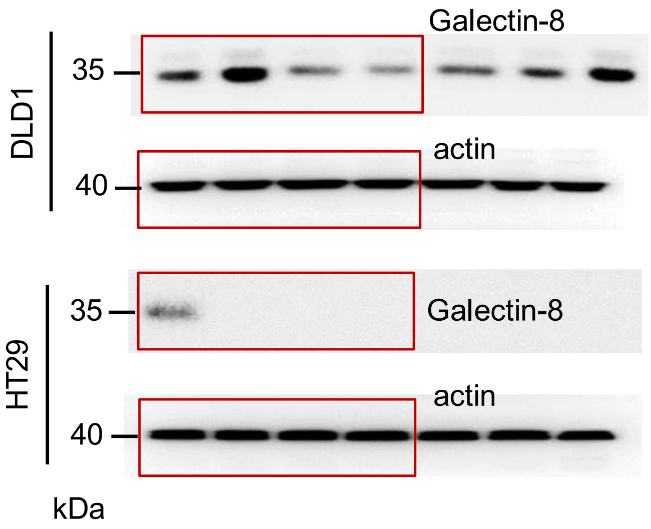

Figure S1E

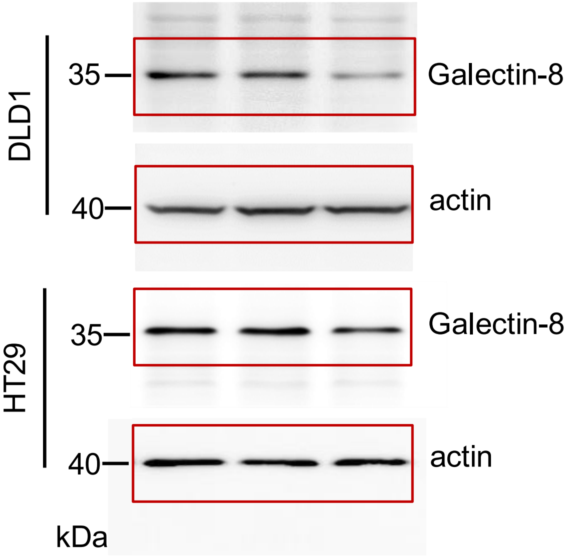

Figure S1F

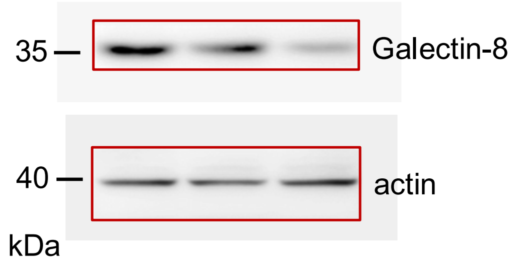

Figure S1G

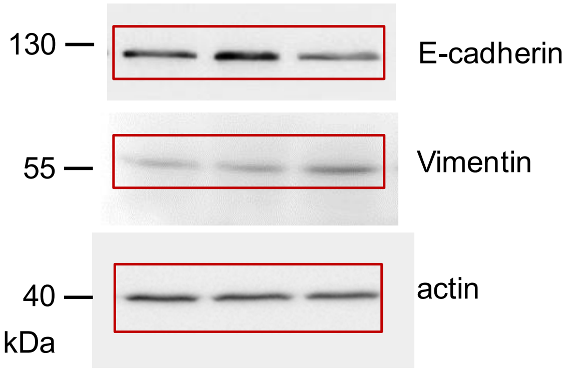

Figure S2D

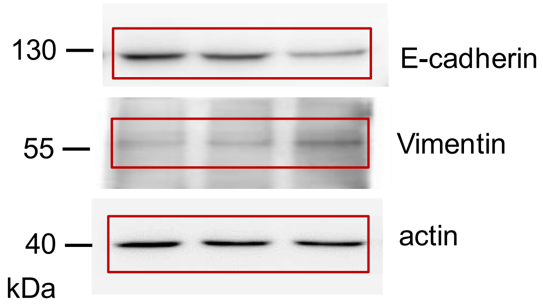

Figure S3B

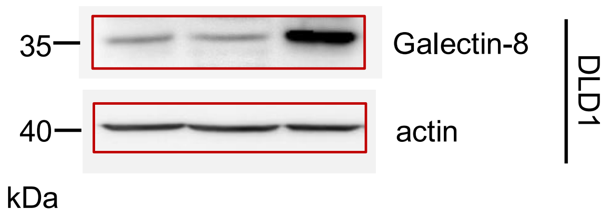

Figure S4G

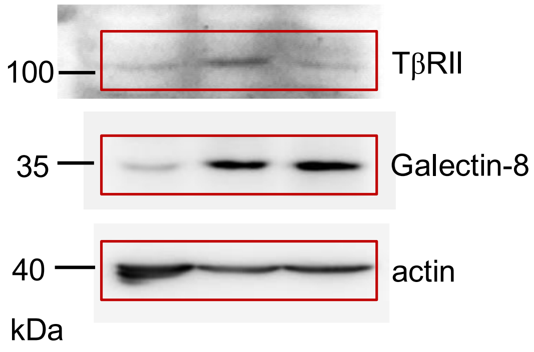

Figure S5C

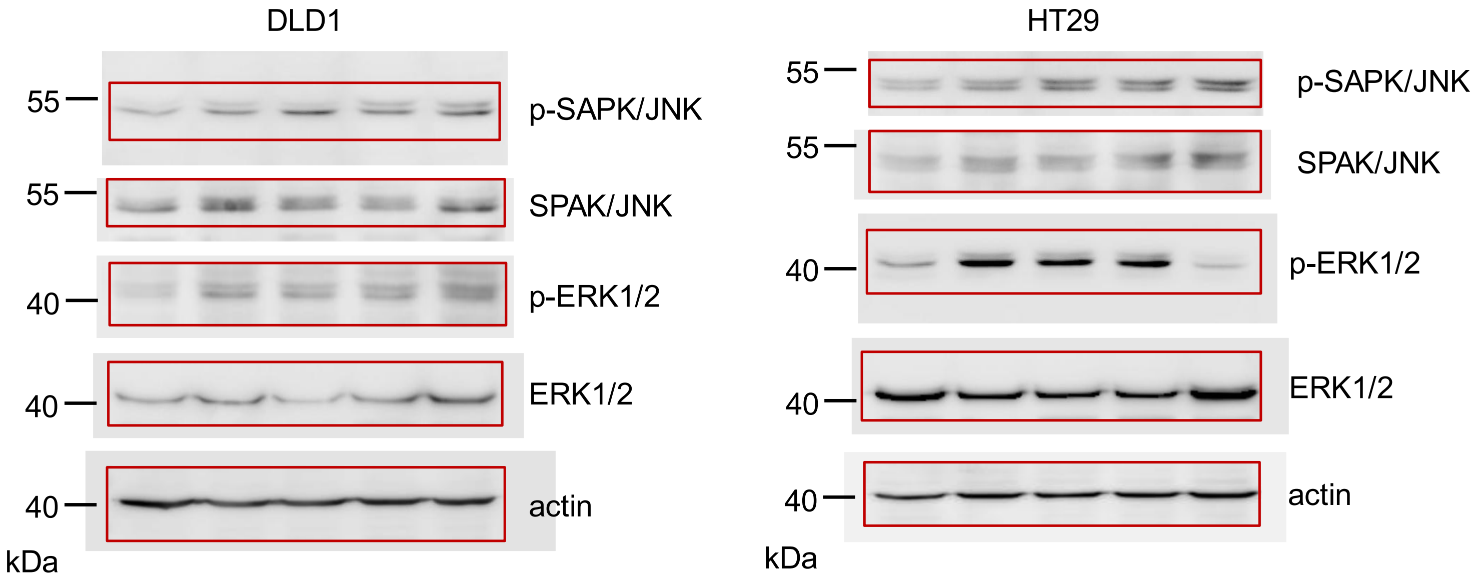
DLD1
HT29
55
p-SAPK/JNK
55
SPAK/JNK
40
p-ERK1/2
40
ERK1/2
40
actin
kDa
55
p-SAPK/JNK
55
SPAK/JNK
40
p-ERK1/2
40
ERK1/2
40
actin
kDa

Figure S6C

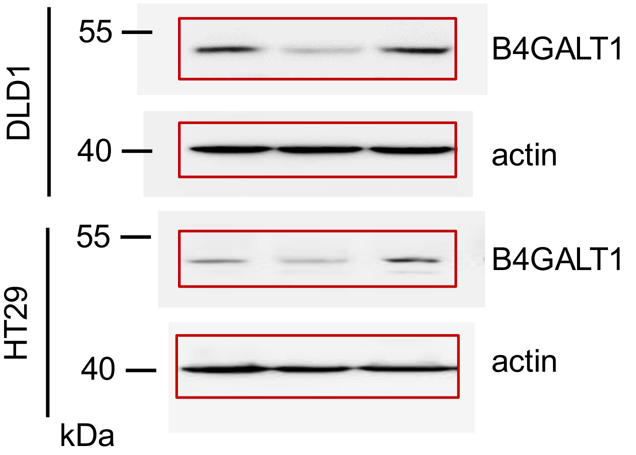

DLD1
55
40
B4GALT1
actin
HT29
55
40
B4GALT1
actin
kDa

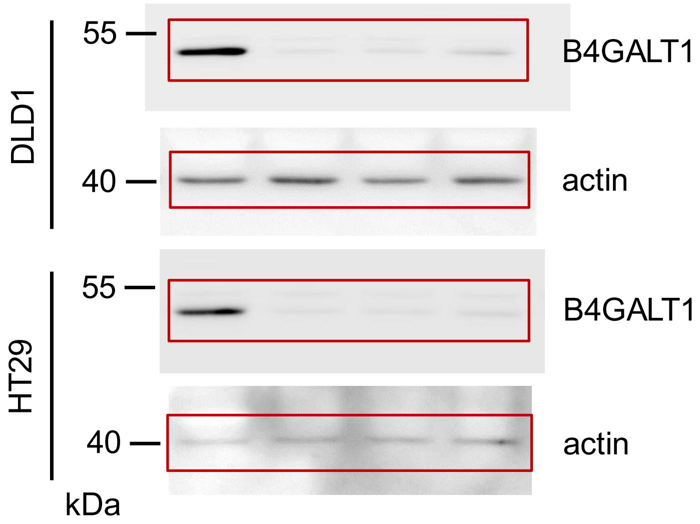

DLD1
55
40
B4GALT1
actin
HT29
55
40
B4GALT1
actin
kDa

Figure S8B

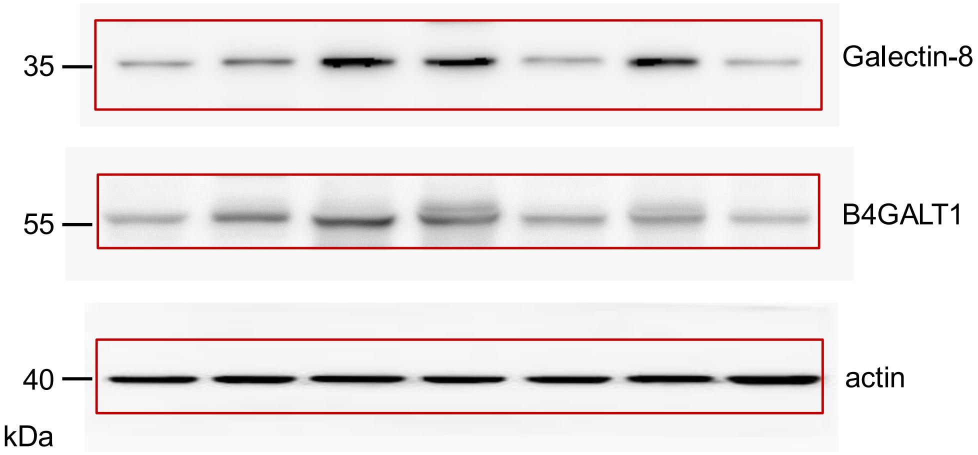

Supplement: Supplementary file 2 — Uncropped westernblot images [file 41419_2024_7028_MOESM2_ESM.pdf]
